# The Effect of Financial Incentives on Quality Measures in the Treatment of Diabetes Mellitus: a Randomized Controlled Trial

**DOI:** 10.1007/s11606-021-06714-8

**Published:** 2021-04-26

**Authors:** Rahel Meier, Corinne Chmiel, Fabio Valeri, Leander Muheim, Oliver Senn, Thomas Rosemann

**Affiliations:** 1 Institute of Primary Care, University of Zurich, Pestalozzistrasse 24, 8091 Zurich, Switzerland; 2 University Hospital Zurich, Zürich, Switzerland

**Keywords:** quality of care, improvement, financial incentives, diabetes mellitus, primary care, spill-over effect

## Abstract

**Background:**

**Financial incentives are often used** to improve quality of care **in chronic care patients.** However, the evidence concerning the effect of financial incentives is still inconclusive.

**Objective:**

To test the effect of financial incentives on quality measures (QMs) in the treatment of patients with diabetes mellitus in primary care. We incentivized a clinical QM and a process QM to test the effect of financial incentives on different types of QMs and to investigate the spill-over effect on non-incentivized QMs.

**Design/Participants:**

Parallel cluster randomized controlled trial based on electronic medical records database involving Swiss general practitioners (GPs). Practices were randomly allocated.

**Intervention:**

All participants received a bimonthly feedback report. The intervention group additionally received potential financial incentives on GP level depending on their performance.

**Main Measures:**

Between-group differences in proportions of patients fulfilling incentivized QM (process QM of annual HbA1c measurement and clinical QM of blood pressure level below 140/95 mmHg) after 12 months.

**Key Results:**

**Seventy-one GPs (median age 52 years, 72% male) from 43 different practices and subsequently 3838 patients with diabetes mellitus (median age 70 years, 57% male) were included.** Proportions of patients with annual HbA1c measurements remained unchanged (intervention group decreased from 79.0 to 78.3%, control group from 81.5 to 81.0%, OR 1.09, 95% CI 0.90–1.32, *p* = 0.39). Proportions of patients with blood pressure below 140/95 improved from 49.9 to 52.5% in the intervention group and decreased from 51.2 to 49.0% in the control group (OR 1.16, 95% CI 0.99–1.36, *p* = 0.06). Proportions of non-incentivized process QMs increased significantly in the intervention group.

**Conclusion:**

GP level financial incentives did not result in more frequent HbA1c measurements or in improved blood pressure control. Interestingly, we could confirm a spill-over effect on non-incentivized process QMs. Yet, the mechanism of spill-over effects of financial incentives is largely unclear.

**Trial Registration:**

ISRCTN13305645

**Supplementary Information:**

The online version contains supplementary material available at 10.1007/s11606-021-06714-8.

## BACKGROUND

Improving quality of care in chronic care patients, especially in patients with diabetes mellitus, has been of major priority around the world.^1^ Different strategies, such as audit and feedback, clinical education, clinical reminders, or financial incentives, have been used, to try to close the gap between actual and optimal care.^2^ Financial incentives, also called pay for performance (P4P) strategies, have been studied in different settings. However, evidence concerning the effect on quality of care is still inconclusive.^3–6^

Many studies investigating the effect of P4P were conducted in the UK, as they pioneered a P4P strategy with its Quality and Outcomes Framework (QOF). Since it was a nationwide pre/post real-life setting without a control group, its effect remains controversial.^7,8^ Randomized controlled trials (RCTs) conducted in the USA or Taiwan showed only modest improvements from P4P interventions,^9–14^ whereas evidence from RCTs in Europe is lacking. In Switzerland, no data on the P4P approach exists and the use of quality measures (QMs), especially in primary care, has been marginal. In Switzerland, general practitioners (GPs) are paid on a fee-for-service basis, based on tariffs contractually agreed between insurers and providers or set by the government.^15^

With the current parallel cluster RCT, we aimed to test whether financial incentives are effective in increasing proportions of patients with diabetes mellitus meeting a QM. Of six diabetes-specific QMs, we incentivized a process QM and a clinical QM to test the effect of incentives on different types of QMs and to investigate spill-over effects on non-incentivized QMs.

## METHODS

### Trial Design

We conducted a parallel cluster RCT with GPs participating in the FIRE network (family medicine International Classification of Primary Care (ICPC) research using electronic medical records (EMRs)).^16^ GPs participating in the FIRE network contribute anonymized patient data to the steadily growing database containing the following data: administrative information, vital signs, laboratory values, medication data, and diagnostic codes, coded in the ICPC-2 classification scheme.^17^

According to the local ethics committee of the canton of Zurich, the project does not fall under the scope of the law on human research and therefore, no ethical consent was necessary (BASEC-Nr. Req-2017-00797). The trial was registered in the ISRCTN registry (identifier: ISRCTN13305645) and the protocol has been published.^18^

### Participants

**Figure 1:**
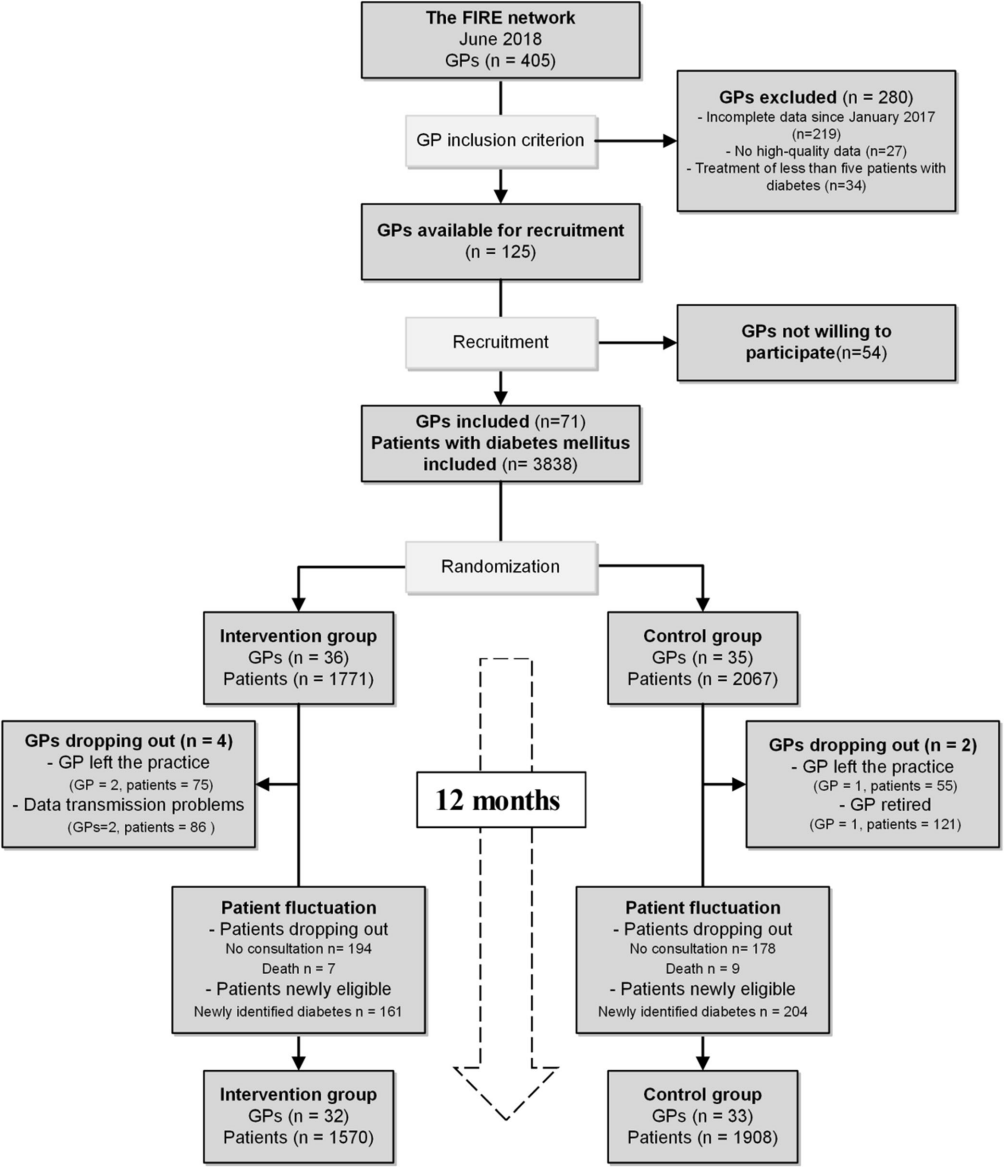
Flowchart of the study including dropouts of GPs and patient fluctuation. GP general practitioner, pat patient.

FIRE GPs had additionally to fulfill the following data availability and data quality criteria to be eligible for the RCT: (a) continuous data delivery since January 2017, (b) delivering HbA1c and blood pressure values in more than 10% of their patients with diabetes mellitus, and (c) treating a minimum of five patients with diabetes mellitus. In June 2018, the eligible GPs received an invitation to participate in the RCT (Fig. 1).

From participating GPs, we included all patients diagnosed with diabetes mellitus if they were diagnosed at least 4 months before the baseline assessment and had at least one consultation during the last 12 months. During the observation period, patients with newly identified diabetes mellitus were included whereas patients not having a consultation within the last 12 month or reported dead dropped out. Patients with diabetes were identified if they met at least one of the following criteria: (1) patients with ICPC-2 codes T89 (insulin-dependent diabetes mellitus) and T90 (insulin-independent diabetes mellitus); (2) patients with antidiabetic medication according to the Anatomical Therapeutic Chemical (ATC) classification system (A10A, A10B, A10X).^19^

### Intervention

Both groups received a bimonthly diabetes feedback report containing information on their patients with diabetes mellitus (age, gender, and body mass index), the proportion of patients receiving at least one HbA1c measurement within the last 12 months, and the proportions of patients with blood pressure measurements and achieving the target blood pressure level. Furthermore, the report contained a key message addressing various issues in the treatment of patients with diabetes mellitus. No patient-specific feedback could be included due to anonymized patient data. An example of the report can be found in Additional File 1. The report was designed to inform GPs about their current achievements in primary outcomes and was used as a sham intervention for the control group. We informed the intervention group at the beginning of the observation period and in a reminder after 6 months about the incentive at GP level. We announced that they will receive an incentive of 75 Swiss francs per improved percentage point in the two QMs mentioned in the feedback report after the observation period. The amount of 75 Swiss francs corresponds to an average net hourly revenue of a Swiss GP.^20^ The control group was blinded for the incentives of the intervention group.

### Outcomes

Primary outcome (two independent primary endpoints): between-group differences in the proportions of patients fulfilling incentivized QMs after 12 months (Table 1).

**Table 1:** Quality Measures Used to Assess Primary and Secondary Outcomes

Type	Measure	Description
Incentivized QMs		
Clinical QM	Blood pressure	Proportion of patients with diabetes mellitus with last blood pressure measurement < 140/85 mmHg in the preceding 12 months.
Process QM	HbA1c	Proportion of patients with diabetes mellitus with at least one measurement of HbA1c in the preceding 12 months.
Non-incentivized QMs		
Process QM	Blood pressure	Proportion of patients with diabetes mellitus with at least one blood pressure measurement in the preceding 12 months.
Clinical QM	HbA1c	Proportion of patients with diabetes mellitus with HbA1c levels < 7.5% in the preceding 12 months.
Process QM	Cholesterol	Proportion of patients with diabetes mellitus with at least one cholesterol measurement in the preceding 12 months.
Clinical QM	Cholesterol	Proportion of patients with diabetes mellitus with total cholesterol < 5 mmol/L in the preceding 12 months.

*QM* quality measures

Secondary outcome: between-group differences in the proportions of patients fulfilling non-incentivized QMs after 12 months (Table 1).

We assessed QM proportions at baseline—based on the 12 months prior to the study start—and every 2 months until the observational period ended 12 months after a GP was included in the study.

### Sample Size

We adjusted the sample size calculation reported in the study protocol, as investigations showed that the assumed baseline proportions have increased. We newly assumed an improvement for the process QM (HbA1c) from currently 80 to 90%. For the clinical QM (controlled blood pressure), we assumed an improvement from currently 60 to 70%. We chose a power of 80% and a type-I error of 5% and we assumed a median number of two GPs per practice and a median number of 30 patients with diabetes mellitus per practice. Furthermore, for all outcomes, we estimated an intraclass correlation at GP level and at patient level based on the FIRE database (for further details see, Additional File 2). Based on these assumptions and a sample size calculation taking the multilevel cluster setting into account, the number of GPs needed for the study remained at 70, hence unchanged to the study protocol.^21^

### Randomization

The randomization took place on practice level to avoid contamination (intervention and control within the same practice). The current proportion of the clinical QM, number of participating GPs per practice, GP network participation, and number of patients with diabetes mellitus were used to stratify randomization. We used the shiny balancer software for randomization^22^ and generated 100 balanced allocation schemes. The settings for the shiny balancer software are displayed in Additional File 1, Table 1. We applied two additional constraints to the generated schemes: (1) the two largest practices were not in the same study group; (2) the difference of number of GPs between the control and intervention group was minimal. We selected one of the proposed schemes randomly.

### Sensitivity Analysis

For a sensitivity analysis, we determined proportions of patients fulfilling the QMs of GPs not willing to participate. The analysis was performed retrospectively for the same time points as for the GPs in the study. With this analysis, we aimed to investigate whether some independent improvements in QMs have occurred simultaneously and if the intervention of solely receiving educational feedback reports had an effect.

### Statistical Methods

We reported categorical data as frequencies and percentages, and continuous variables as means and standard deviations (SD) or median and interquartile range (IQR), as appropriate. To assess the effect of financial incentives, we used hierarchical multivariable logistic regression models, with random variables of patients nested in GPs and GPs nested in practice. The QM fulfillment was the dependent variable, and independent variables were time and group allocation, for which we allowed interaction. Furthermore, we adjusted for age and gender of the GPs and the volume of patients with diabetes mellitus per GP. The same model was used for the sensitivity analysis, with the non-participating GPs as a third group allocation. To visualize trend and effect, we computed for each GP the percentage of patients fulfilling the QMs and aggregated these according to group allocation and time and computed the mean and the Wald 95% confidence interval.

## RESULTS

### Study Population

Of the 125 GPs identified as eligible and invited to participate, 71 gave consent to participate. Due to difficulties in recruitment, 61 GPs started in September 2018 in cohort 1, whereas 10 GPs were enrolled in a second cohort starting in November 2018. Randomization took place at the practice level (intervention group: 21 practices and 36 GPs, control group: 22 practices and 35 GPs). Subsequently, all their patients with diabetes mellitus were included, resulting in a starting population of 3838 patients (intervention group: 1771 patients, control group: 2067 patients). During the 12-month observation period, four GPs dropped out in the intervention group (161 patients) and two in the control group (176 patients). Reasons are outlined in Figure 1 and information on dropout date of GPs is available in Additional File 1, Table 2. Patient fluctuation due to changes in eligibility status (death, no consultation within 12 months, or newly identified patients with diabetes mellitus) was minimal and more than 85% of patients were observed during the entire observation period (for exact numbers, see Fig. 1). The intervention phase ended as planned after 12 months.

**Table 2:** Baseline Characteristics by Group

	Intervention group	Control group
Practice and GP characteristics		
Practices	21	22
GPs	36	35
GPs per practice	1.0 (1.0–2.0)	1.0 (1.0–2.0)
Network participation (no)	4 (11.1)	2(5.7)
Age	48.0 (42.0–57.3)	54.0 (46.0–63.0)
Gender (male)	24 (66.7)	27 (77.1)
Volume of patients with DM per GP (%)	5.0 (3.3–6.4)	4.9 (3.7–6.4)
Patient characteristics		
Patients	1771	2067
Patient age	70.0 (59.0–79.0)	69.0 (60.0–77.0)
Patient gender (male)	989 (55.8)	1209 (58.6)
Treatment and disease characteristics		
Consultations in observation period	8 (3–15)	7 (3–12)
Blood pressure measurements	3 (1–4)	2 (1–4)
Systolic blood pressure [mm Hg]	137.5 (128.4–149.0)	134.0 (125.0–143.3)
Diastolic blood pressure [mm Hg]	80.0 (74.0–86.0)	80.0 (73.3–85.0)
HbA1c measurements	2 (1–3)	3 (1–4)
HbA1c [%]	6.8 (6.3–7.5)	6.8 (6.3–7.5)
Cholesterol measurement	1 (1–2)	1 (1–1)
Cholesterol [mmol/L]	4.5 (3.8–5.3)	4.6 (3.8–5.4)
BMI measurements	2 (1–3)	2 (1–3)
BMI [kg/m^2^]	29.4 (26.5–33.1)	29.6 (26.3–33.5)
DM associated comorbidities		
Obesity	759 (42.9)	855 (41.4)
Hypertension	1566 (88.4)	1765 (85.4)
Hyperlipidemia	1098 (62.0)	1167 (56.5)
Chronic kidney disease	212 (12.0)	242 (11.7)
Peripheral arterial disease	54 (3.0)	80 (3.9)
Coronary heart disease	127 (7.2)	107 (5.2)
Heart failure	48 (2.7)	35 (1.7)
Stroke	29 (1.6)	38 (1.8)
Retinopathy	11 (0.6)	3 (0.1)
Neuropathy	44 (2.5)	56 (2.7)
DM associated medication		
Insulin only	159 (9.0)	129 (6.2)
Oral anti-diabetic medication only	866 (48.9)	1112 (53.8)
Combination therapy insulin and oral anti-diabetic medication	258 (14.6)	262 (12.7)
Antihypertensive medication	862 (48.7)	975 (47.2)
Antiplatelet therapy and anticoagulants	736 (41.6)	806 (39.0)
Lipid-lowering medication	258 (14.6)	262 (12.7)

Data are presented as *n*, *n* (%), or median (interquartile range), unless otherwise stated. *GP* general practitioner, *DM* diabetes mellitus, *BMI* body mass index. Identification schemes for diseases can be found in Additional File 1, Table 3

At baseline, GPs had a median age of 52 years (IQR: 44–60), 72% were male and 91.5% worked in a group practice. The patients had a median age of 70 years (IQR: 60–78), 57% were male. Table 2 gives detailed information on the study population including group comparison. The median incentive payed to the GPs in the intervention group was 637.50 Swiss francs (IQR: 300–1200) (€: 603.-, IQR: 284–1137).

### Primary Outcomes

**Figure 2:**
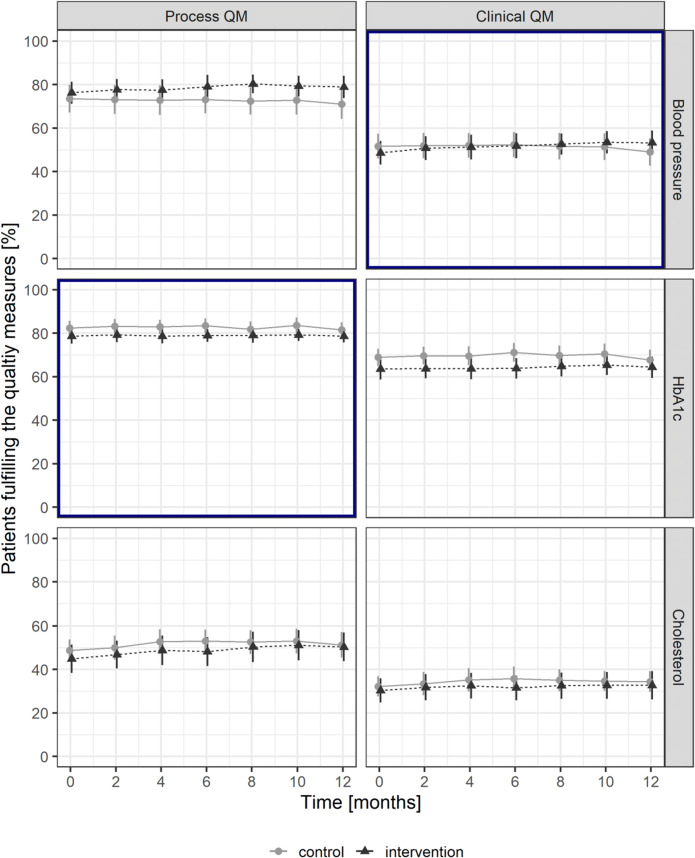
Proportions of patients fulfilling the quality measures during the observation period. Primary outcomes are surrounded by thick frames. Illustrated are mean and Wald 95% confidence interval for each group. QM quality measure.

After 12 months, the proportions decreased for the process QM HbA1c from 79.0 to 78.3% for the intervention group and from 81.5 to 81.0% for the control group (Fig. 2). The proportions of patients achieving a blood pressure target level below 140/85 mmHg improved from 49.9 to 52.5% for the intervention group, and decreased from 51.2 to 49.0% for the control group (Fig. 2). The odds ratio for the interactive effect of time and intervention over the entire observation period was 1.09 (95% CI: 0.90–1.32, *p* = 0.39) for the process QM of HbA1c and 1.16 (95% CI: 0.99–1.36, *p* = 0.06) for the clinical QM of achieving a blood pressure target level below 140/85 mmHg (Table 3). The detailed results of the logistic regression, and the estimates of the hierarchical random effects for GPs and GP nested in practices, can be found in Additional File 1, Table 4 and Table 5.

**Table 3:** Interactive Effect of Time and Intervention over the Entire Observation Period

Type	Subject	OR	95% CI	*p* value
Primary outcomes				
Clinical QM	Blood pressure	1.16	0.99–1.36	0.06
Process QM	HbA1c	1.09	0.90–1.32	0.39
Secondary outcomes				
Process QM	Blood pressure	1.24	1.02–1.50	<0.05
Clinical QM	HbA1c	1.15	0.97–1.35	0.11
Process QM	Cholesterol	1.17	1.00–1.38	<0.05
Clinical QM	Cholesterol	1.06	0.90–1.25	0.47

The model is adjusted for age and gender of the GPs and the volume of patients with diabetes mellitus per GP. *QM* quality measure, *OR* odds ratio, *CI* confidence interval

### Secondary Outcomes

After 12 months, the proportions in all four non-incentivized QMs increased slightly for the intervention group, whereas for the control group, only the proportions for cholesterol QMs increased (Fig. 2). The logistic regression analysis revealed that the intervention had a significant spill-over effect on the two process QMs of the secondary outcomes (blood pressure: OR 1.24, 95% CI 1.02–1.50, *p* < 0.05, cholesterol: OR 1.17, 95% CI 1.00–1.38, *p* > 0.05) (Table 3).

### Sensitivity Analysis

For the sensitivity analysis, we included 33 GPs from 16 different practices with 1541 patients with diabetes mellitus. Practice, GP, and patient characteristics were similar to the study population (Additional File 1, Table 6), whereas baseline proportions were lower for non-participants regarding blood pressure QMs (Additional File 1, Fig. 1). The results of the logistic regressions showed that there was no study independent improvement occurring during the observational period (Additional File 1, Table 7) and that the educational feedback report had no effect.

## DISCUSSION

In this parallel cluster RCT, we evaluated the effect of financial incentives on QMs in the treatment of patients with diabetes mellitus in primary care. We tested financial incentives targeted at the GP level in combination with feedback reports, in comparison to feedback reports only. Financial incentives did not have a significant effect on primary outcomes; the proportion of patients receiving annual HbA1c measurements and the proportion of patients achieving the recommended blood pressure target level was stable. Some effects were observed on secondary outcomes.

Systematic reviews^3,4^ concluded that financial incentives targeting process QMs, which can directly be altered by providers, showed greater effects than financial incentives targeting clinical QMs, which can only be influenced indirectly. In contrast, in this study, we detected no significant effect of the financial incentives on the directly incentivized process and clinical QM. However, in case of the process QM of measuring HbA1c annually, for which rates of 80% were achieved, we suspect a ceiling effect, and therefore, no further improvement was attained. Measuring HbA1c is the most standard procedure in diabetes mellitus care and other quality initiatives already taking place in the Swiss health care environment might have led to such already high-performance levels at baseline^23,24^ and notably the fee-for-service setting where the setting itself already gives an incentives for regular measurement. However, other European countries report achievement rates of over 90%,^25^ implying that higher rates are not beyond reach. The appearance of a ceiling effect for HbA1c measures is also supported by our models, which reveal smaller variability of random effects on practice and GP level for HbA1c, than for the other QMs. Furthermore, the presence of ceiling effects is supported when comparing to the achievement rates of 2014 where lower achievement rates but higher variation was observed.^26^

Despite we observed no effect in directly incentivized QMs, we observed some effects on non-incentivized process QMs. It is known that spill-over effects of financial incentives on non-incentivized QM can occur, generally with smaller effect sizes than in incentivized QMs.^27^ Spill-over effects might indicate that GPs’ awareness on the condition improved and that GPs addressed a more holistic disease approach.^28^ Hysong et al. also reported that financial incentives improved care documentation without necessarily improving the care provided. Despite these plausible mechanisms of spill-over effects, our finding should be treated with caution, as the effect on directly incentivized QMs—presumably due to ceiling effects—was limited and other unknown confounders might matter.

Our results show that the potential of financial incentives to increase proportions of patients fulfilling a QM might be limited, and that feedback reporting with educational aspects only had no effect. First, the promised incentive of 75 Swiss francs per percentage point improvement was rather low. With an assumed effect size, the amount achieved would have been slightly less than 1% of the annual fee-for-service income of a Swiss GP. Compared to the QOF, where GPs received financial incentives of up to 25% of their income,^29^ our incentive was most likely too low to have a major effect. Second, P4P initiatives and feedback reporting are complex and behavioral economic principles and the design aspects play an important role. Factors such as the amount of the incentive, incentive frequencies, and the level of payment have an influence on the effectiveness of incentives.^30–32^ Feedback reports are found to be most effective when they are actionable and individualized and when specific goals are set.^33,34^

### Strengths and Limitations of This Study

To our knowledge, this is the first cluster RCT testing a P4P strategy in diabetes care within Europe. In general, quality improvement studies in diabetes care are of importance, due to high prevalence and disease burden. We were able to implement a simple P4P intervention and to blind the control group. Additionally, we were able to compare the study results with another cohort not participating in the study. This study therefore closes a gap, left open by the many observational studies. Compared to the nationwide-conducted and governmentally authorized intervention in the UK, we were only able to test our intervention with a comparably small number of GPs, however large enough to validly detect potential effects.

The major limitation of this study is its inherent risk of selection bias as GPs participate on a voluntary basis in the FIRE project. However, GPs participating in the FIRE project cover 8% of GPs working in the German-speaking region of Switzerland^35^ but they are most likely GPs more engaged in research and quality of care, than their counterparts not participating in the FIRE project.^36^ Additionally, using EMR favors higher quality.^37,38^ However, EMRs are only used by around 70% of GPs in Switzerland^39^ but an EMR is a key requirement for participating in the FIRE project.

In the baseline analysis of this RCT, we conducted further investigations to disentangle the complex interplay of different factors influencing quality of care, also including patient characteristics.^40^ However, the influence of available explanatory variables of practice, GP, and patient level on performance was surprisingly small. Another potential explanatory variable—not systematically retrieved—is the availability of specifically trained chronic care nurses. To our knowledge, only one practice had a special trained nurse, since in Switzerland it is highly uncommon that trained nurses work in GP practices.

Further limitations arose due to the underlying database. Such databases are known to be prone to missing data and data quality issues. These issues might not be apparent at baseline due to randomization. However, we cannot preclude that the increases in proportions of patients fulfilling process QMs are due to better data reporting, and not due to enhanced care delivery. This confounder might especially be of concern in the process QM of blood pressure measurement, as it is very likely that the blood pressure values, which need to be entered manually, are not exclusively reported in the intended field. However, cross-validation indicated high validity of the database.^41^ Lastly, limited knowledge was available about death of patients, as only very few GPs code it appropriately to be visible for FIRE. To overcome this limitation, patients with no consultation within the last 12 months were excluded due to loss of follow-up.

## CONCLUSION

GP level financial incentives did not result in more frequent HbA1c measurements or in improved blood pressure control. Interestingly, we observed spill-over effects on non-incentivized process QMs. Yet, the mechanism of spill-over effects of financial incentives is largely unclear. In general, however, the potential of financial incentives to improve the quality of diabetes treatment may be limited. In order to potentially maximize the impact of financial incentives, behavioral economic principles should be given greater consideration.

## Supplementary Information

ESM 1 (DOC 218 kb)

ESM 2 (PDF 1231 kb)

ESM 3 (DOCX 399 kb)

## Data Availability

The data are gathered within the ongoing FIRE project. The FIRE database can be accessed at any time by the scientific team of the institute. For external requests, access has to be requested from the head of the institute.
