# Supplementary material for: The Effect of Financial Incentives on Quality Measures in the Treatment of Diabetes Mellitus: a Randomized Controlled Trial
Source: J Gen Intern Med. 2021 Apr 26;37(3):556–64. doi: 10.1007/s11606-021-06714-8 (PMC8858366; doi:10.1007/s11606-021-06714-8)
Supplement: Supplementary file 2 — (PDF 1231 kb) [file 11606_2021_6714_MOESM2_ESM.pdf]

Sample size of P4P

27 April, 2018

- Quantity structure/Distributions
  - Tables
  - Graphs
- Eligibility 1
  - Excluded practices
- Analysis of process QI to exclude data transfer problems
- Distribution of clinical QI
- Eligibility 2
  - Blood pressure
  - HbA1c
  - Cholesterol
- Eligibility 3
  - Eligibility depending on cut limit (absolute numbers)
  - Eligibility depending on cut limit (percentage)
- Tables of ρ
  - ρ of process QI according to cut limit
  - ρ with process QI cut <0.1 and according to number of levels
  - ρ of clinical QI according to cut limit
  - ρ of clinical QI with process QI cut <0.1 and according to number of levels
- Graph of ρ
  - Graph ρ of process QI
  - Graph σ^2 of process QI
  - Graph ρ of clinical QI
  - Graph ρ of clinical QI
- Graph of random effects of PI and CI with PI cut < 0.1
  - Random effects of BP
  - Random effects of HbA1c
  - Random effects of Cholesterol
- Sample size computation
  - Dateset
  - Formula
    - Glossary
    - Sample size of one level (one sided)
    - Dilution bias
    - Variance inflation factor VIF_3
    - Sample size
  - Statistical parameters
  - ρ_s, ρ_e and σ^2_{total}
    - Process QI
    - Clinical QI
  - Number of patients per GP (n_e) and number of GPs per praxis (n_s)
  - Sample size with $n_s = 2.0$ and $n_e = 10$
  - Sample size with $n_s = 2.0$ and $n_e = 20$
  - Sample size with $n_s = 2.0$ and $n_e = 30$
  - Sample size with $n_s = 2.5$ and $n_e = 10$
  - Sample size with $n_s = 2.5$ and $n_e = 20$
  - Sample size with $n_s = 2.5$ and $n_e = 30$
- Sample size plots
- Comments
  - Why ρ_s and ρ_e are used instead of ICC_2 and ICC_1?
  - Why *power.prop.test* and not *SSizeLogisticCon* was used for N_0
  - Why σ_{pat} has value 3.29?
  - Alternative methods to compute sample size

Quantity structure/Distributions

Tables

Eligibel patient with

- at least 8 month consultations
- at least 5 patients per praxis

Number of practices	65
Number of GP	157

Number of patients	5,751

	Mean	Median
GP per practices	2.4	2
Patient per practices	88.5	75
Patiente per GP	36.6	26

Graphs

Distribution of practices according to number of month with consultation

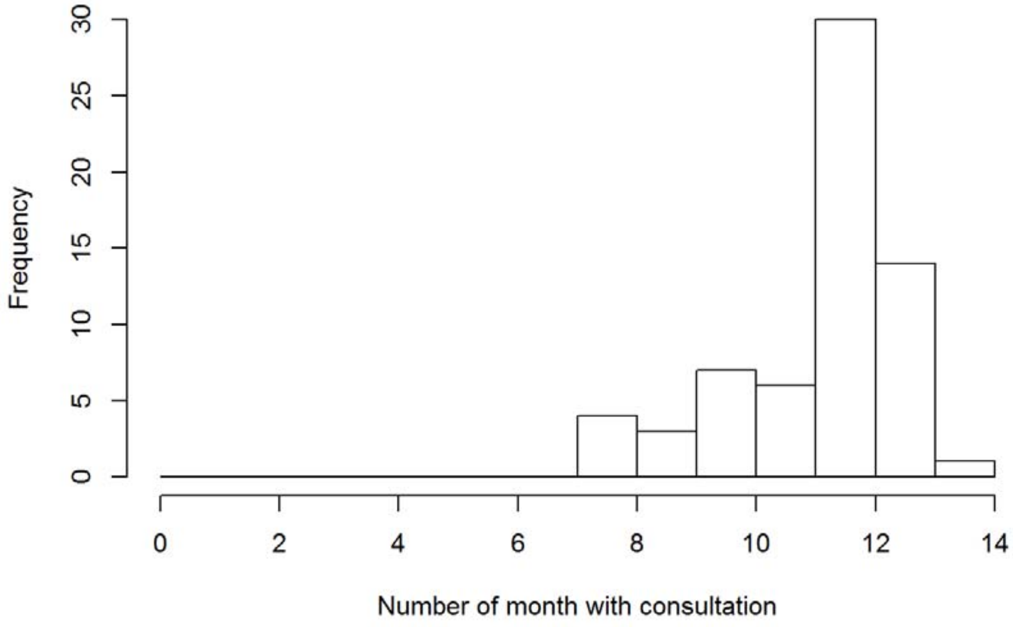

Distribution of practices according to patients

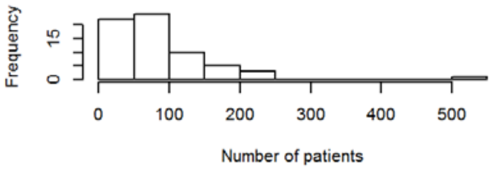

Distribution of practices according to patients (N patient ≤ 50)

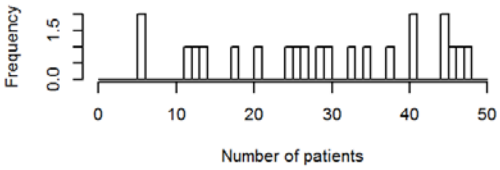

Distribution of GP according to patient

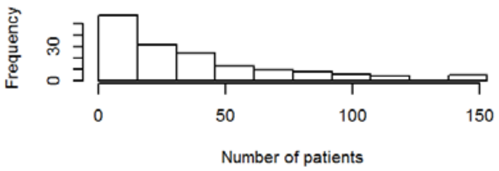

Distribution of GP according to patients (N patient ≤ 20)

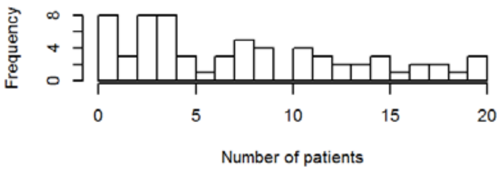

Distribution of practices according to GP

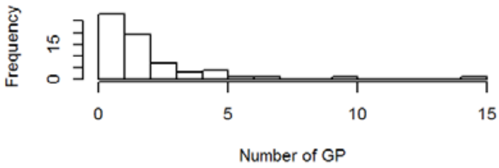

Eligibility 1

	N praxis	N GP	N patients	% praxis	% GP	% patients
Patient with diabetes	69	166	5,926	100.0	100.0	100.0
At least 8 month consultations	67	159	5,757	97.1	95.8	97.1
At least 5 patients per praxis	65	157	5,751	94.2	94.6	97.0

Excluded practices

Practice ID	N GP	N Diabetes patient	Consultation Month	Process QI (%)	Outcome QI (%)
102	3	46	6	41.3	30.4
138	1	4	12	75.0	0.0
149	1	2	8	100.0	100.0
200	4	123	6	42.3	22.8

Analysis of process QI to exclude data transfer problems

Low process QI indicates problems during transfer of data. Cut limits for process QI are defined to exclude praxis with data problems.

- The blue vertical lines in the following figures are arbitrarly choosen cut levels to illustrate how cutting affect the sample.
- Solid and dashed red lines are the means, respectively, the medians.
- Green lines are assumed and target values defined in the proposal.

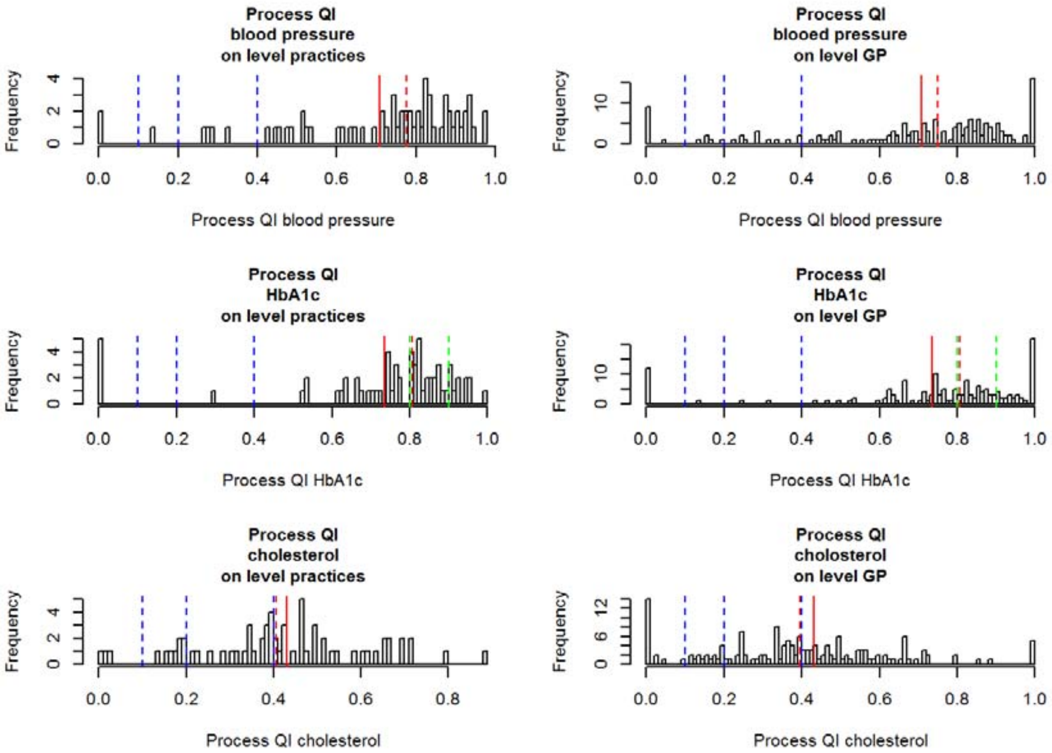

Distribution of clinical QI

- The red solid and dashed vertical lines are the means, respectively, medians
- The green lines the supposed and targed % of QI defined in the proposal.

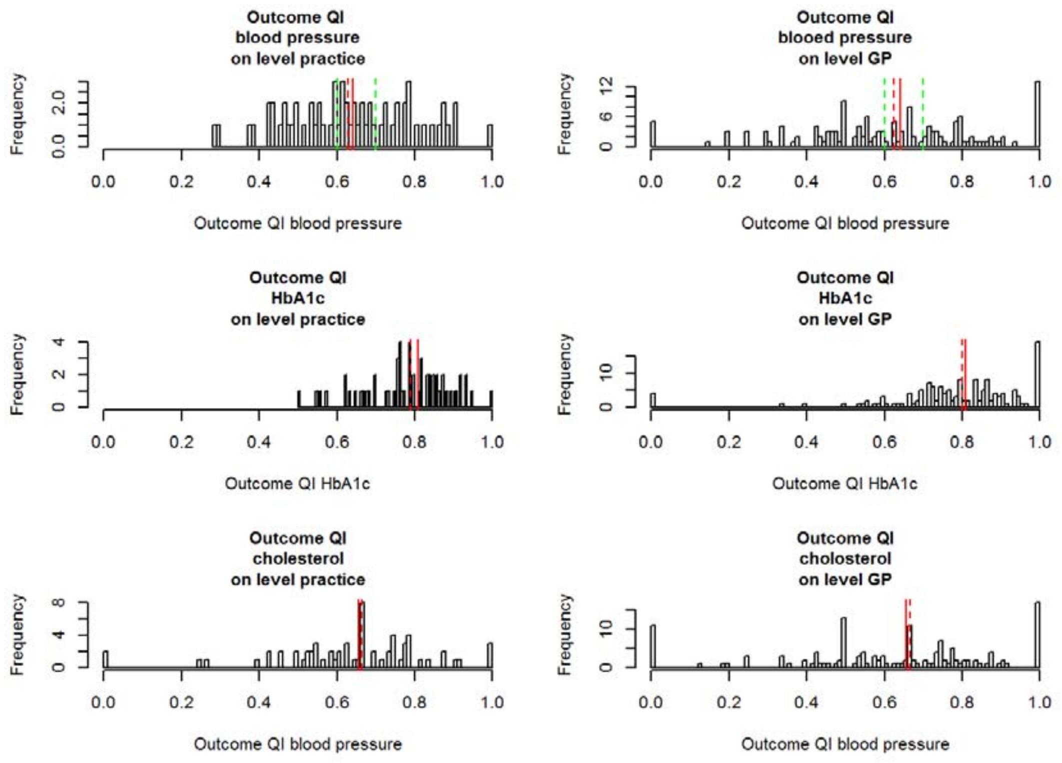

Eligibility 2

Blood pressure

Cut limit	N Practice	N GP	N Patient	% Practice	% GP
No cut	65	157	5,751	100.0	100.0
<0.1	63	154	5,711	96.9	98.1
<0.2	62	152	5,681	95.4	96.8
<0.4	58	144	5,323	89.2	91.7

HbA1c

Cut limit	N Practice	N GP	N Patient	% Practice	% GP
No cut	65	157	5,751	100.0	100.0
<0.1	60	148	5,367	92.3	94.3
<0.2	60	148	5,367	92.3	94.3
<0.4	59	146	5,337	90.8	93.0

Cholesterol

Cut limit	N Practice	N GP	N Patient	% Practice	% GP
No cut	65	157	5,751	100.0	100.0
<0.1	62	152	5,596	95.4	96.8
<0.2	55	136	5,112	84.6	86.6
<0.4	36	90	3,564	55.4	57.3

Eligibility 3

Blood pressure and HbA1c are the primary outcomes. The study population must consider only praxis which take account for all exclusion criteria of both parameter. This table shows how sample size change if the process QI criteria is considered for both parameters.

Eligibility depending on cut limit (absolute numbers)

	N Practice BP	N Practice HbA1c	N Practice BP+HbA1c	N GP BP	N GP HbA1c	N GP BP+HbA1c	N Patient BP	N Patient HbA1c	N Patient BP+HbA1c
no cut	65	65	65	157	157	157	5,751	5,751	5,751
<0.1	63	60	58	154	148	145	5,711	5,367	5,327
0.2	62	60	57	152	148	143	5,681	5,367	5,297
0.4	58	59	55	144	146	140	5,323	5,337	5,157

Eligibility depending on cut limit (percentage)

	% Practice BP	% Practice HbA1c	% Practice BP+HbA1c	% GP BP	% GP HbA1c	% GP BP+HbA1c	% Patient BP	% Patient HbA1c	% Patient BP+HbA1c
no cut	100.0	100.0	100.0	100.0	100.0	100.0	100.0	100.0	100.0
<0.1	96.9	92.3	89.2	98.1	94.3	92.4	99.3	93.3	92.6
0.2	95.4	92.3	87.7	96.8	94.3	91.1	98.8	93.3	92.1
0.4	89.2	90.8	84.6	91.7	93.0	89.2	92.6	92.8	89.7

Tables of ρ

ρ of process QI according to cut limit

Parameter	Levels	σ^2_{total}	σ^2_{pract}	σ^2_{gp}	σ^2_{pat}	ρ_{gp}	ρ_{pat}
BP	3	5.05	1.01	0.75	3.29	0.58	0.35
	3 and cut < 0.1	4.69	0.68	0.72	3.29	0.49	0.30
	3 and cut < 0.2	4.60	0.60	0.71	3.29	0.46	0.28
	3 and cut < 0.4	4.32	0.39	0.64	3.29	0.38	0.24
HBA1C	3	6.09	2.45	0.35	3.29	0.88	0.46
	3 and cut < 0.1	3.79	0.17	0.33	3.29	0.35	0.13
	3 and cut < 0.2	3.79	0.17	0.33	3.29	0.35	0.13
	3 and cut < 0.4	3.73	0.13	0.32	3.29	0.28	0.12
CHOL	3	4.15	0.44	0.42	3.29	0.51	0.21
	3 and cut < 0.1	3.87	0.17	0.41	3.29	0.29	0.15
	3 and cut < 0.2	3.73	0.00	0.44	3.29	0.00	0.12
	3 and cut < 0.4	3.64	0.04	0.31	3.29	0.12	0.10

ρ with process QI cut <0.1 and according to number of levels

Parameter	Levels	σ^2_{total}	σ^2_{pract}	σ^2_{gp}	σ^2_{pat}	ρ_{gp}	ρ_{pat}
BP	3	4.69	0.68	0.72	3.29	0.49	0.30
	2-praxis	4.35	1.06	NA	3.29	0.24	NA
	2-GP	4.57	NA	1.28	3.29	NA	0.28
HBA1C	3	3.79	0.17	0.33	3.29	0.35	0.13

Parameter	Levels	σ^2_{total}	σ^2_{pract}	σ^2_{gp}	σ^2_{pat}	ρ_{gp}	ρ_{pat}
	2-praxis	3.68	0.39	NA	3.29	0.11	NA
	2-GP	3.77	NA	0.48	3.29	NA	0.13
CHOL	3	3.87	0.17	0.41	3.29	0.29	0.15
	2-praxis	3.77	0.48	NA	3.29	0.13	NA
	2-GP	3.86	NA	0.57	3.29	NA	0.15

ρ of clinical QI according to cut limit

Parameter	Level and cut limit	σ^2_{total}	σ^2_{pract}	σ^2_{gp}	σ^2_{pat}	ρ_{gp}	ρ_{pat}
BP	3	3.74	0.36	0.10	3.29	0.79	0.12
	3 and cut < 0.1	3.74	0.36	0.10	3.29	0.79	0.12
	3 and cut < 0.2	3.74	0.36	0.10	3.29	0.79	0.12
	3 and cut < 0.4	3.76	0.37	0.10	3.29	0.79	0.12
HBA1C	3	3.51	0.12	0.10	3.29	0.53	0.06
	3 and cut < 0.1	3.51	0.12	0.10	3.29	0.53	0.06
	3 and cut < 0.2	3.51	0.12	0.10	3.29	0.53	0.06
	3 and cut < 0.4	3.51	0.11	0.10	3.29	0.52	0.06
CHOL	3	3.60	0.13	0.18	3.29	0.43	0.09
	3 and cut < 0.1	3.60	0.13	0.17	3.29	0.43	0.09
	3 and cut < 0.2	3.62	0.14	0.19	3.29	0.41	0.09
	3 and cut < 0.4	3.62	0.10	0.24	3.29	0.30	0.09

ρ of clinical QI with process QI cut <0.1 and according to number of levels

Parameter	Levels	σ^2_{total}	σ^2_{pract}	σ^2_{gp}	σ^2_{pat}	ρ_{gp}	ρ_{pat}
BP	3	3.74	0.36	0.10	3.29	0.79	0.12
	2-praxis	3.71	0.42	NA	3.29	0.11	NA
	2-GP	3.74	NA	0.45	3.29	NA	0.12
HBA1C	3	3.51	0.12	0.10	3.29	0.53	0.06
	2-praxis	3.48	0.19	NA	3.29	0.06	NA
	2-GP	3.50	NA	0.21	3.29	NA	0.06
CHOL	3	3.60	0.13	0.17	3.29	0.43	0.09
	2-praxis	3.53	0.24	NA	3.29	0.07	NA
	2-GP	3.62	NA	0.33	3.29	NA	0.09

Graph of ρ

Graph ρ of process QI

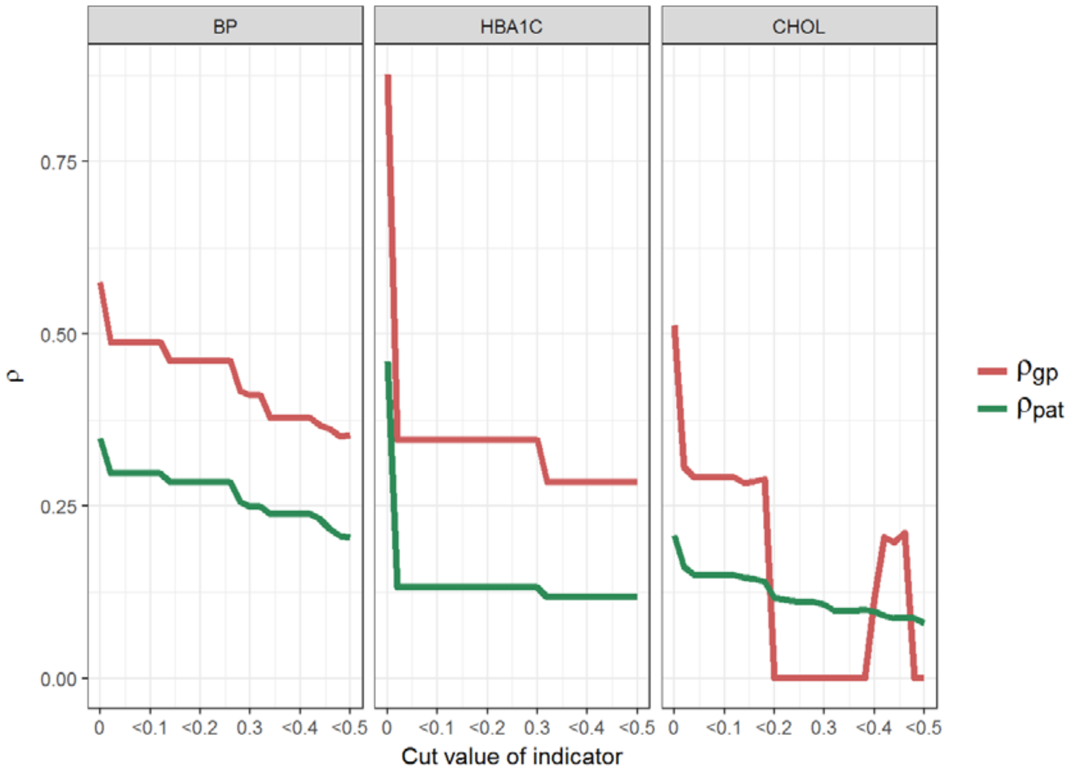

Graph σ^2 of process QI

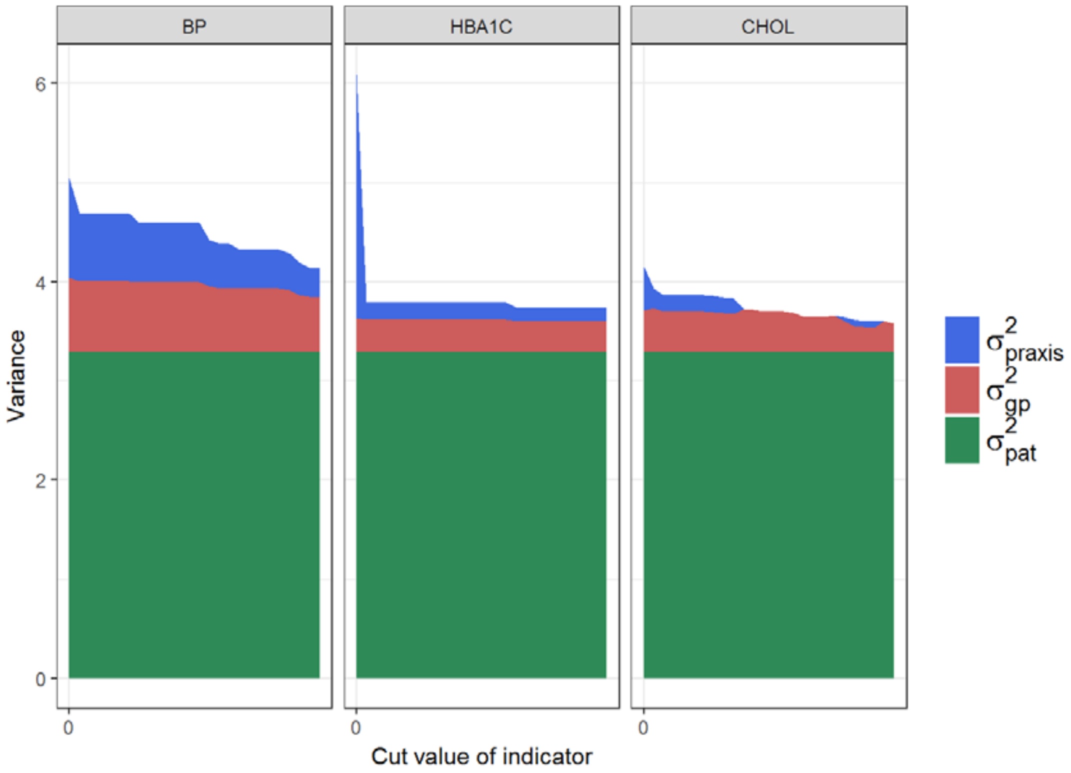

Graph ρ of clinical QI

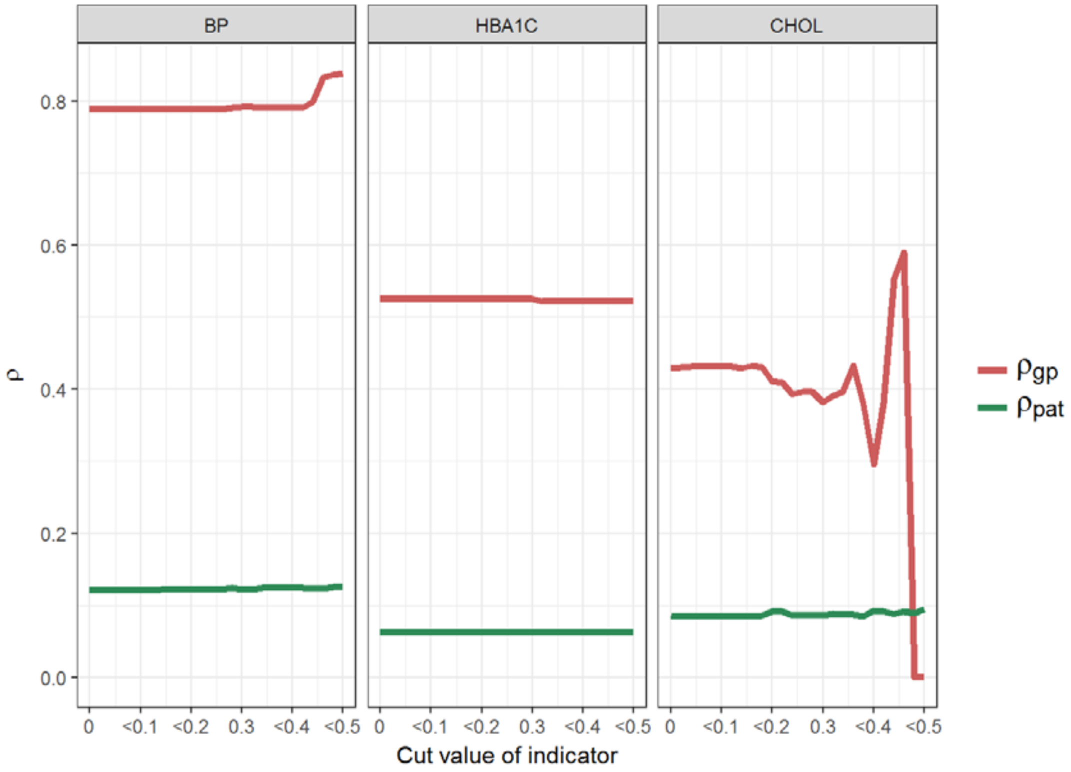

Graph ρ of clinical QI

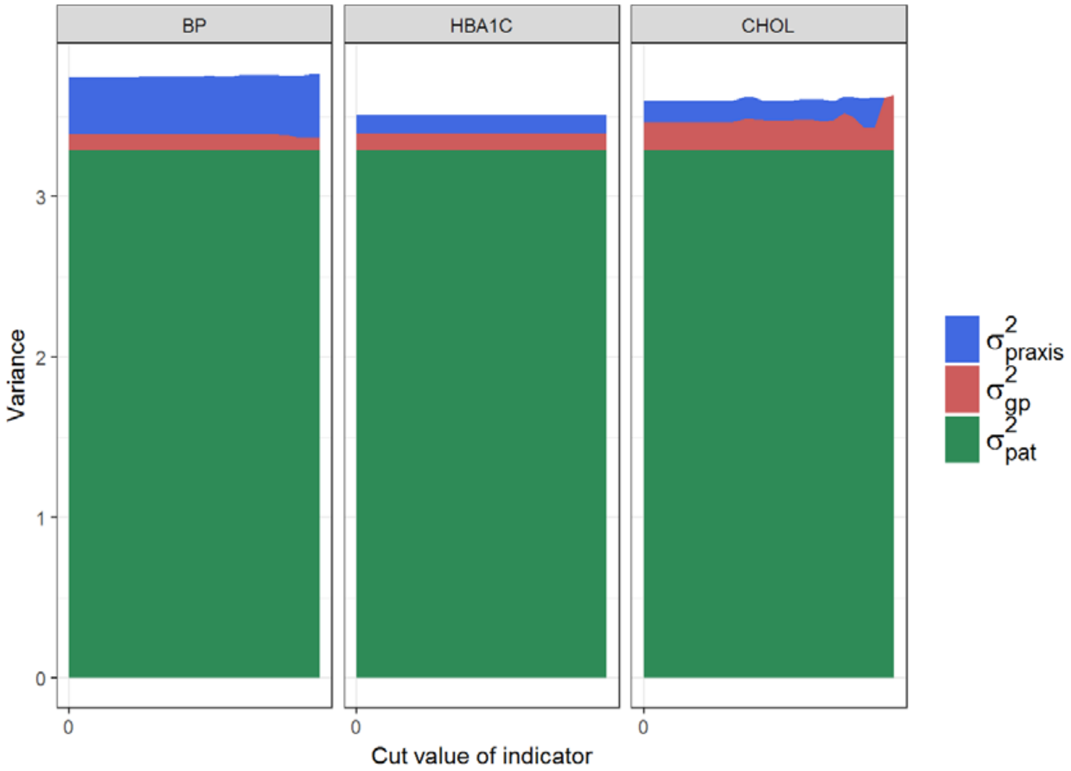

Graph of random effects of PI and CI with PI cut < 0.1

Random effects of BP

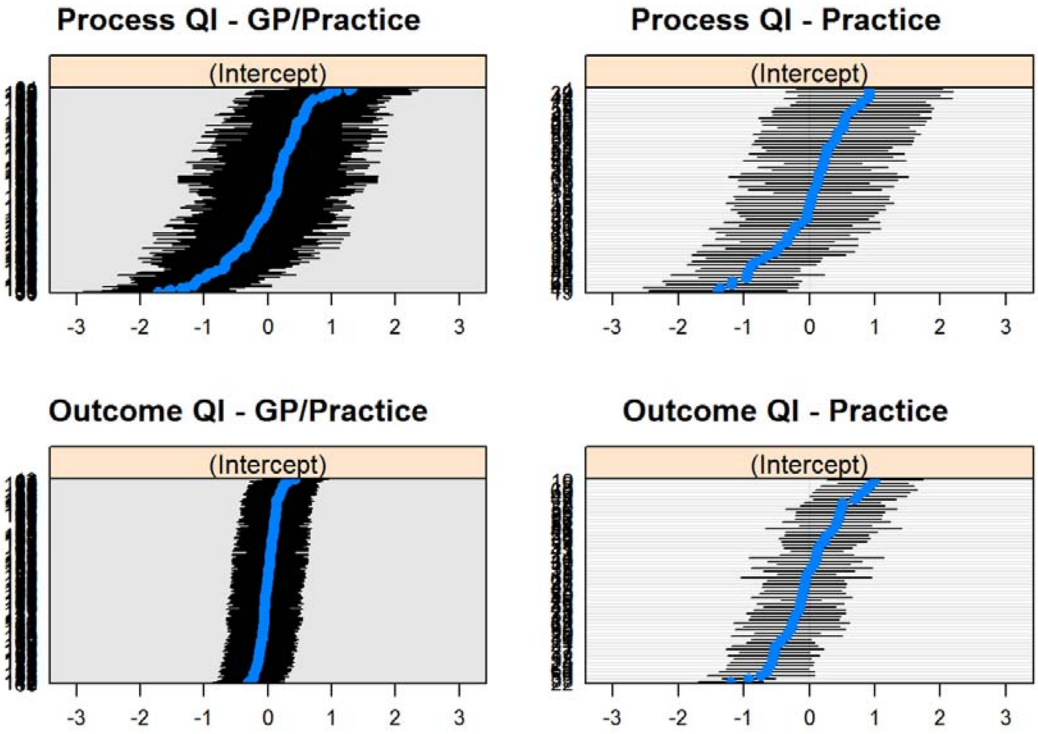

Random effects of HbA1c

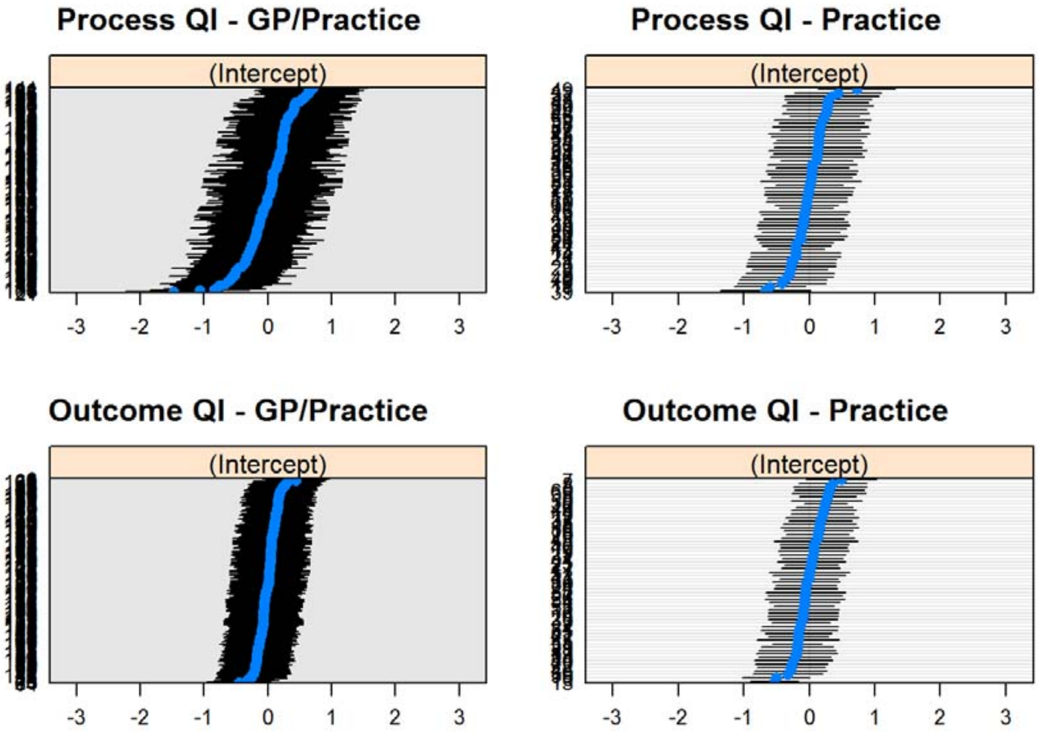

Random effects of Cholesterol

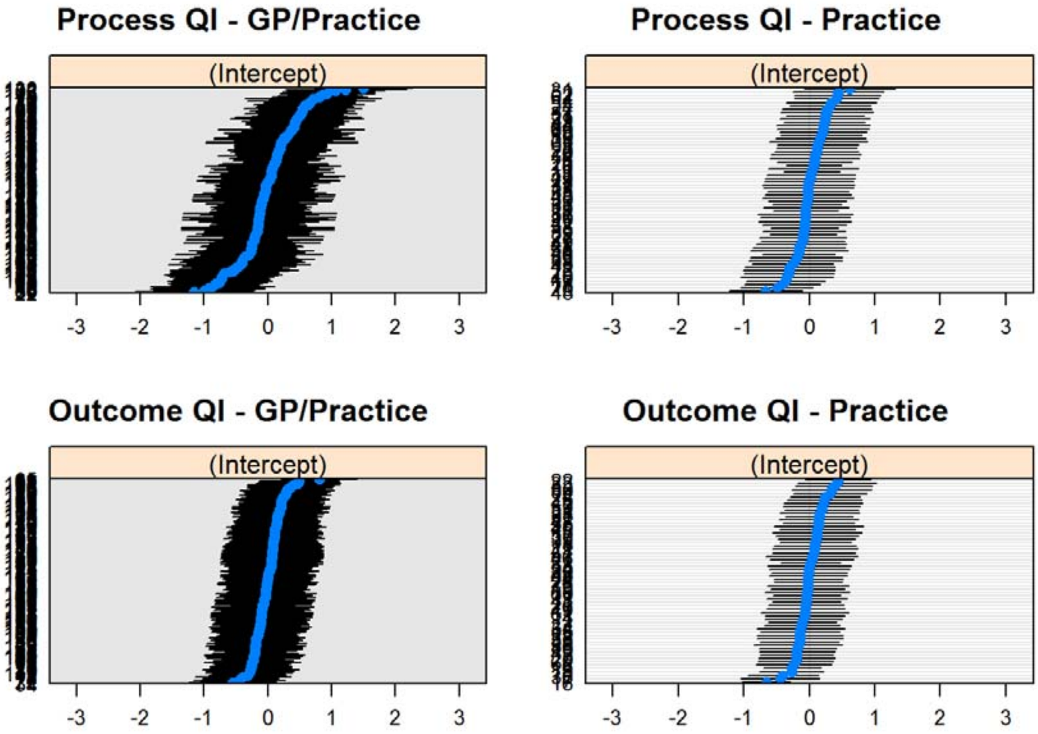

Sample size computation

Dateset

Based on cutting level < 0.1.

Formula

Lists of formula relevant for sample size computation of a 3 level regression analysis.

Literatur: Tereenstra(2008): Sample size calculations for 3-level cluster randomized trials

Glossary

- $n_e = N_{pat/GP}$: Number of patient within GP (evaluation, first level)
- $n_s = N_{GP/praxis}$: Number of GP within praxis (subject, second level)
- $n_c = N_{praxis}$: Number of praxis (cluster, third level)

- σ_e^2: Variance between patient within GP (between **e**valuations within the same **s**ubject)
- σ_s^2: Variance between GP within praxis (between **s**ubjects within the same **c**luster)
- σ_c^2: Variance between praxis (between clusters)
- σ^2: is $\sigma_c^2 + \sigma_s^2 + \sigma_e^2$. variance of a single patient (**e**valuation score)

- $ICC_2 = \frac{\sigma_c^2}{\sigma_c^2+\sigma_s^2+\sigma_e^2}$ intraclass correlations at the second level (**s**ubject)
- $ICC_1 = \frac{\sigma_s^2}{\sigma_c^2+\sigma_s^2+\sigma_e^2}$ intraclass correlations at the first level (**e**valuation)

- $\rho_s(n_e) = \frac{ICC_2}{ICC_1+ICC_2} = \frac{\sigma_c^2}{\sigma_c^2+\sigma_s^2}$: pairwise correlation between mean scores of any two subjects within the same cluster (GP within praxis)
- $\rho_e = ICC_1 + ICC_2 = \frac{\sigma_c^2+\sigma_s^2}{\sigma_c^2+\sigma_s^2+\sigma_e^2}$: pairwise correlation between any two evaluations within the same subject (patient within GP)

- VIF: Variance inflation factor

Sample size of one level (one sided)

$N_0 = 2(z_\alpha + z_\beta)^2 \cdot \frac{\sigma^2}{\delta^2}$ $N_0 = 2(z_\alpha + z_\beta)^2 \cdot \frac{p_1(1-p_1)+p_2(1-p_2)}{(p_1-p_2)^2}$ (Ahn, p. 18)

Dilution bias

> For small n_e, the sampling error in the mean scores will decrease the estimated correlation (a phenomenon akin to regression dilution bias [8]) and this can be quantified:

$\rho_s(n_e) = \rho_s \cdot w$

$w = \frac{n_e \rho_e}{1 + (n_e - 1)\rho_e}$

Variance inflation factor VIF_3

$VIF3 = [1 + (n_e - 1)\rho_e) \cdot [1 + (n_s - 1)\rho_s w]$

Sample size

$n_c n_s n_e = VIF_3 N_0$

Statistical parameters

Statistical parameter	Value
Number of levels	3
Power	80%
Significance level	5%
Direction	one sided

ρ_s, ρ_e and σ^2_{total}

Process QI

Levels	σ^2_{total}	ρ_{gp}	ρ_{pat}	
BP	3	4.69	0.49	0.30
HBA1C	3	3.79	0.35	0.13
CHOL	3	3.87	0.29	0.15

Clinical QI

Parameter	Levels	σ^2_{total}	ρ_{gp}	ρ_{pat}
BP	3	3.74	0.79	0.12
HBA1C	3	3.51	0.53	0.06
CHOL	3	3.60	0.43	0.09

Number of patients per GP (n_e) and number of GPs per praxis (n_s)

- We define $n_s = 2.0$ and $n_s = 2.5$. These are the median and mean of GP/praxis (see table above).
- We define $n_e = 10$, $n_e = 20$ and $n_e = 30$. These are the third and two third of the mean and the mean (see table above).

The number of cluster is computed with: $n_c = \frac{VIF_3 N_0}{n_s n_e}$

Sample size with $n_s = 2.0$ and $n_e = 10$

Parameter	Type	σ^2_{tot}	σ^2_{pract}	σ^2_{GP}	σ^2_{pat}	ρ_{gp}	ρ_{pat}	p1 (%)	p2 (%)	Diff (%)	$N_{GP/praxis}$	$N_{patient/GP}$	w	VIF_3	N_0	N_{pat}	N_{GP}	N_{pract}
BP	proc	4.69	0.68	0.72	3.29	0.49	0.30	50	60	10	2	10	0.90	5.14	305	1,567	157	78
	clin	3.74	0.36	0.10	3.29	0.79	0.12	60	70	10	2	10	0.97	3.04	280	853	85	43

Parameter	Type	σ^2_{tot}	σ^2_{pract}	σ^2_{GP}	σ^2_{pat}	ρ_{gp}	ρ_{pat}	p1 (%)	p2 (%)	Diff (%)	$N_{GP/praxis}$	$N_{patient/GP}$	w	VIF_3	N_0	N_{pat}	N_{GP}	N_{pract}
HBA1C	proc	3.79	0.17	0.33	3.29	0.35	0.13	80	90	10	2	10	0.84	2.64	157	414	41	21
	clin	3.51	0.12	0.10	3.29	0.53	0.06	50	60	10	2	10	0.92	1.89	305	577	58	29
CHOL	proc	3.87	0.17	0.41	3.29	0.29	0.15	50	60	10	2	10	0.80	2.78	305	849	85	42
	clin	3.60	0.13	0.17	3.29	0.43	0.09	50	60	10	2	10	0.88	2.13	305	651	65	33

Sample size with $n_s = 2.0$ and $n_e = 20$

Parameter	Type	σ^2_{tot}	σ^2_{pract}	σ^2_{GP}	σ^2_{pat}	ρ_{gp}	ρ_{pat}	p1 (%)	p2 (%)	Diff (%)	$N_{GP/praxis}$	$N_{patient/GP}$	w	VIF_3	N_0	N_{pat}	N_{GP}	N_{pract}
BP	proc	4.69	0.68	0.72	3.29	0.49	0.30	50	60	10	2	20	0.89	9.57	305	2,919	146	73
	clin	3.74	0.36	0.10	3.29	0.79	0.12	60	70	10	2	20	0.73	5.20	280	1,459	73	36
HBA1C	proc	3.79	0.17	0.33	3.29	0.35	0.13	80	90	10	2	20	0.75	4.42	157	692	35	17
	clin	3.51	0.12	0.10	3.29	0.53	0.06	50	60	10	2	20	0.57	2.85	305	868	43	22
CHOL	proc	3.87	0.17	0.41	3.29	0.29	0.15	50	60	10	2	20	0.78	4.72	305	1,438	72	36
	clin	3.60	0.13	0.17	3.29	0.43	0.09	50	60	10	2	20	0.65	3.35	305	1,023	51	26

Sample size with $n_s = 2.0$ and $n_e = 30$

Parameter	Type	σ^2_{tot}	σ^2_{pract}	σ^2_{GP}	σ^2_{pat}	ρ_{gp}	ρ_{pat}	p1 (%)	p2 (%)	Diff (%)	$N_{GP/praxis}$	$N_{patient/GP}$	w	VIF_3	N_0	N_{pat}	N_{GP}	N_{pract}
BP	proc	4.69	0.68	0.72	3.29	0.49	0.30	50	60	10	2	30	0.93	14.01	305	4,272	142	71
	clin	3.74	0.36	0.10	3.29	0.79	0.12	60	70	10	2	30	0.80	7.37	280	2,065	69	34
HBA1C	proc	3.79	0.17	0.33	3.29	0.35	0.13	80	90	10	2	30	0.82	6.20	157	971	32	16
	clin	3.51	0.12	0.10	3.29	0.53	0.06	50	60	10	2	30	0.67	3.80	305	1,160	39	19
CHOL	proc	3.87	0.17	0.41	3.29	0.29	0.15	50	60	10	2	30	0.84	6.65	305	2,028	68	34
	clin	3.60	0.13	0.17	3.29	0.43	0.09	50	60	10	2	30	0.74	4.57	305	1,394	46	23

Sample size with $n_s = 2.5$ and $n_e = 10$

Parameter	Type	σ^2_{tot}	σ^2_{pract}	σ^2_{GP}	σ^2_{pat}	ρ_{gp}	ρ_{pat}	p1 (%)	p2 (%)	Diff (%)	$N_{GP/praxis}$	$N_{patient/GP}$	w	VIF_3	N_0	N_{pat}	N_{GP}	N_{pract}
BP	proc	4.69	0.68	0.72	3.29	0.49	0.30	50	60	10	2.5	10	0.81	5.86	305	1,788	179	72
	clin	3.74	0.36	0.10	3.29	0.79	0.12	60	70	10	2.5	10	0.58	3.52	280	986	99	39
HBA1C	proc	3.79	0.17	0.33	3.29	0.35	0.13	80	90	10	2.5	10	0.60	2.87	157	450	45	18
	clin	3.51	0.12	0.10	3.29	0.53	0.06	50	60	10	2.5	10	0.40	2.06	305	627	63	25
CHOL	proc	3.87	0.17	0.41	3.29	0.29	0.15	50	60	10	2.5	10	0.64	3.00	305	915	92	37
	clin	3.60	0.13	0.17	3.29	0.43	0.09	50	60	10	2.5	10	0.48	2.32	305	707	71	28

Sample size with $n_s = 2.5$ and $n_e = 20$

Parameter	Type	σ^2_{tot}	σ^2_{pract}	σ^2_{GP}	σ^2_{pat}	ρ_{gp}	ρ_{pat}	p1 (%)	p2 (%)	Diff (%)	$N_{GP/praxis}$	$N_{patient/GP}$	w	VIF_3	N_0	N_{pat}	N_{GP}	N_{pract}
BP	proc	5	1	1	3	0.49	0.30	50	60	10	2.5	20	0.89	11.03	305	3,363	168	67

Parameter	Type	σ^2_{tot}	σ^2_{pract}	σ^2_{GP}	σ^2_{pat}	ρ_{gp}	ρ_{pat}	p1 (%)	p2 (%)	Diff (%)	$N_{GP/praxis}$	$N_{patient/GP}$	w	VIF_3	N_0	N_{pat}	N_{GP}	N_{pract}
	clin	4	0	0	3	0.79	0.12	60	70	10	2.5	20	0.73	6.16	280	1,726	86	35
HBA1C	proc	4	0	0	3	0.35	0.13	80	90	10	2.5	20	0.75	4.88	157	764	38	15
	clin	4	0	0	3	0.53	0.06	50	60	10	2.5	20	0.57	3.18	305	969	48	19
CHOL	proc	4	0	0	3	0.29	0.15	50	60	10	2.5	20	0.78	5.15	305	1,572	79	31
	clin	4	0	0	3	0.43	0.09	50	60	10	2.5	20	0.65	3.72	305	1,135	57	23

Sample size with $n_s = 2.5$ and $n_e = 30$

Parameter	Type	σ^2_{tot}	σ^2_{pract}	σ^2_{GP}	σ^2_{pat}	ρ_{gp}	ρ_{pat}	p1 (%)	p2 (%)	Diff (%)	$N_{GP/praxis}$	$N_{patient/GP}$	w	VIF_3	N_0	N_{pat}	N_{GP}	N_{pract}
BP	proc	4.69	0.68	0.72	3.29	0.49	0.30	50	60	10	2.5	30	0.93	16.19	305	4,937	165	66
	clin	3.74	0.36	0.10	3.29	0.79	0.12	60	70	10	2.5	30	0.80	8.80	280	2,466	82	33
HBA1C	proc	3.79	0.17	0.33	3.29	0.35	0.13	80	90	10	2.5	30	0.82	6.88	157	1,078	36	14
	clin	3.51	0.12	0.10	3.29	0.53	0.06	50	60	10	2.5	30	0.67	4.30	305	1,310	44	17
CHOL	proc	3.87	0.17	0.41	3.29	0.29	0.15	50	60	10	2.5	30	0.84	7.30	305	2,228	74	30
	clin	3.60	0.13	0.17	3.29	0.43	0.09	50	60	10	2.5	30	0.74	5.12	305	1,563	52	21

Sample size plots

Plots with selected parameter variations. Parameters are hold constand, respectively:

- $\rho_e = 0.3$
- $\rho_s = 0.3$ without weighting. Weighting is considered in sample size computation
- $\delta = 0.1$
- $Power = 0.8$
- $n_s = 3$
- direction test: one sided

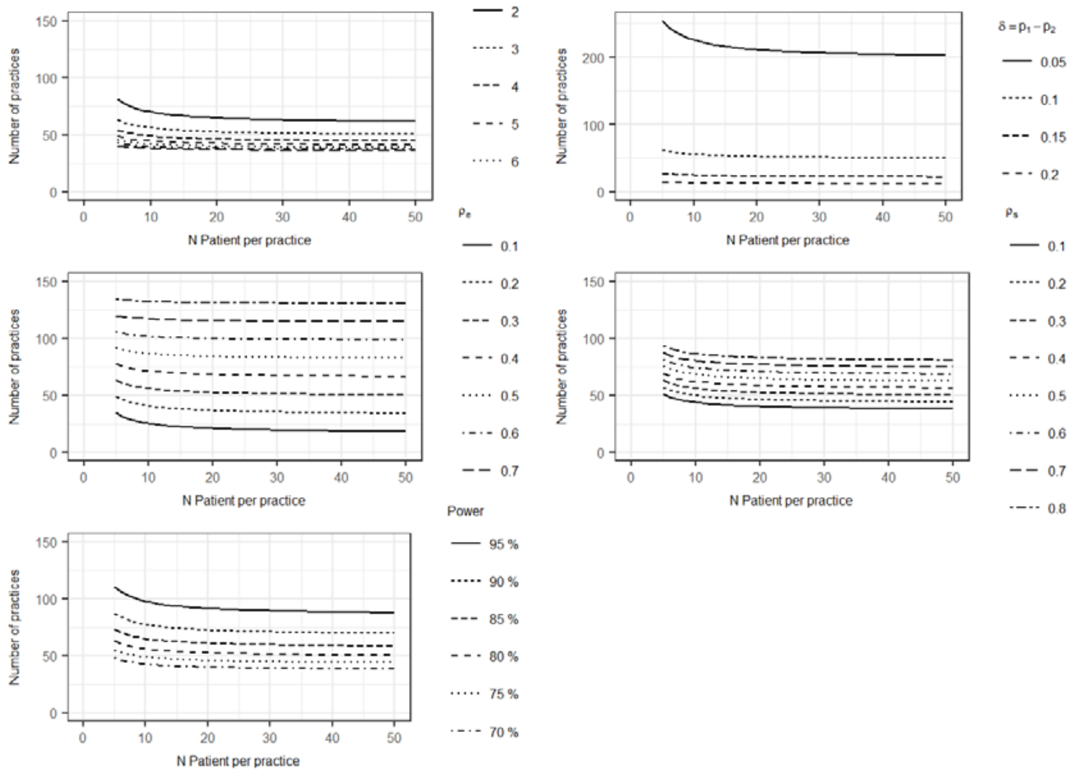

Comments

Why ρ_s and ρ_e are used instead of ICC_2 and ICC_1?

ICC reports the proportion of variance explained for each grouping level that is the one level variance related to total variance. The relevant ICC (named ρ) to compute sample size is the cumulated variance of the upper levels of the level of interest related to the cumulated variance of the upper levels and level of interest:

1. $\rho_s = \frac{\sigma_c^2}{\sigma_c^2+\sigma_s^2}$
2. $\rho_e = \frac{\sigma_c^2+\sigma_s^2}{\sigma_c^2+\sigma_s^2+\sigma_e^2}$

This makes sense: The lower the variance of the level of interest, the higher the correlation. For the two cases above:

1. The level of interest is subject (correlation between subject within cluster). If subject within cluster is hight correlated then either the variance of the cluster is very high compared to the variance of the subject or the variance of the subject is very low compared to the variance of the cluster.
2. The level of interest is evaluation (correlation between evaluation within subject). If evaluation within subject within cluster is very hight then the variance is very low compared to the sum of the variance of cluster and subject.

- $\rho_s(n_e) = \frac{ICC_2}{ICC_1+ICC_2} = \frac{\sigma_c^2}{\sigma_c^2+\sigma_s^2}$: pairwise correlation between mean scores of any two subjects within the same cluster (GP within praxis)
- $\rho_e = ICC_1 + ICC_2 = \frac{\sigma_c^2+\sigma_s^2}{\sigma_c^2+\sigma_s^2+\sigma_e^2}$: pairwise correlation between any two evaluations within the same subject (patient within GP)

Why *power.prop.test* and not *SSizeLogisticCon* was used for N_0

SSizeLogisticCon_ from library *powerMediation* was discovered after this work. *SSizeLogisticCon* gives a much lower sample size, about 50% less. It seems that odds ratio needs lower sample size to detect the correspondent difference of proportion.

```
p1=0.5
p2=0.6
(or <- (p2/(1-p2))/(p1/(1-p1)))
```

```
## [1] 1.5
```

```
power.prop.test(p1=0.5, p2=0.6, sig.level=0.05, power=0.8, alternative="two.sided")$n
```

```
## [1] 387.3385
```

```
SSizeLogisticCon(p1=p1, OR=or,alpha=0.05, power=0.8)
```

```
## [1] 191
```

Why σ_{pat} has value 3.29?

This is the variance of the logistic distribution (https://en.wikipedia.org/wiki/Logistic_distribution) which is equal to $\frac{\pi^2}{3}$.

Alternative methods to compute sample size

See chapter 8: Size Matters … Just no in the Way That You Think. In More Statistical and Methodogical Myths and Urbend Legends (2015).
