# Supplementary material for: The Effect of Financial Incentives on Quality Measures in the Treatment of Diabetes Mellitus: a Randomized Controlled Trial
Source: J Gen Intern Med. 2021 Apr 26;37(3):556–64. doi: 10.1007/s11606-021-06714-8 (PMC8858366; doi:10.1007/s11606-021-06714-8)
Supplement: Supplementary file 3 — (DOCX 399 kb) [file 11606_2021_6714_MOESM3_ESM.docx]

Example of an educational diabetes feedback report (translated) – GPs received the German original


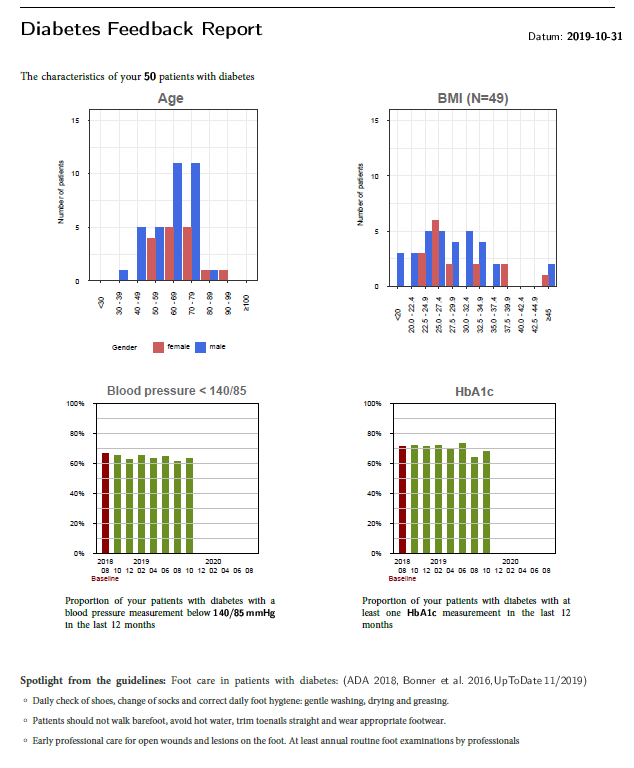


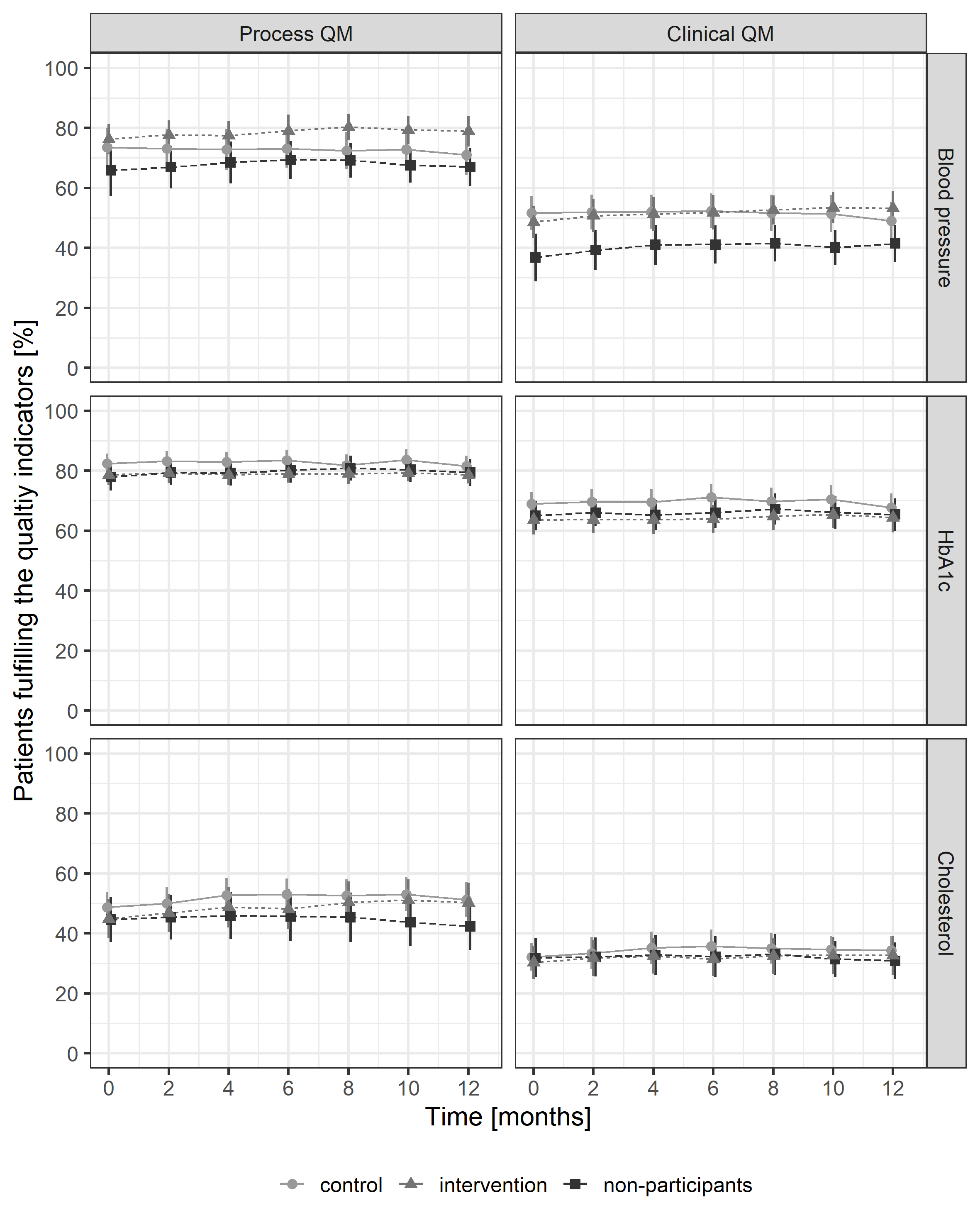


Figure 1 Illustration of sensitivity analysis: proportions of patients fulfilling the quality measures during the observation period. Primary outcomes are surrounded by thick frames. Illustrated are mean and Wald-95%-confidence-interval for each group. QM: quality measure

Supplementary table 1: Settings of the shiny balancer

| Setting | Value |
| --- | --- |
| Imbalance measure logical | 1 |
| Weight logical | 1 |
| Imbalance measure character | 1 |
| Weight character | 1 |
| Imbalance measure integer | 1 |
| Weight integer | 1 |
| Imbalance measure double | 5 |
| Weight double | 1 |
| Generator seed | 2 |
| Random choice seed | 2 |
| Allocation ratio | 0.5 |
| Number of generated schemes | 1000 |
| Unique schemes | 1 |
| Imbalance limit | 0.63 |
| Number of balanced schemes | 100 |

Supplementary table 2: Length of observation period of GPs dropping out:

|  | **Reason for drop out** | **Length of observation period** | **Number of patients dropping out** |
| --- | --- | --- | --- |
| **Intervention group** | GP left the practice (n= 2) | 6 month observation period | 33 patients |
|  | GP left the practice (n= 2) | 8 month observation period | 42 patients |
|  | Data transmission problems (n=2) | 2 month observation period | 52 patients |
|  | Data transmission problems (n=2) | 2 month observation period | 34 patients |
| **Control group** | GP left the practice (n= 1) | 8 month observation period | 55 patients |
|  | GP retired (n= 1) | 2 month observation period | 121 patients |

Supplementary table 3: Disease identification schemes

| **Disease** | **Identification scheme** |
| --- | --- |
| Obesity | BMI ≥30 (last 12 months)  OR When BMI not available AND ICPC = T82 |
| Hypertension | ATC = 'C02\|C03A\|C03EA01\|C0[78]\|C09[AB]'  OR  ICPC = K85\|K86\|K87  OR two blood pressure measurement ≥ 140/85 |
| Hyperlipidaemia | ICPC = T93  OR  ATC = ‘C10A’  OR  Triglyceride >1.7 mmol/l  OR  Total-Cholesterol >5 mmol/l OR  LDL-Cholesterol > 3 mmol/l OR  (sex = 'female' AND HDL-Cholesterol ≤1.2 mmol/l) OR  (sex = 'male' AND HDL-Cholesterol ≤1 mmol/l) |
| Chronic kidney disease stadium ≥ 3b | When last GFR < 45 ml/min and if there is at least one GFR<90 ml/min between date of last measure - 3 month and date of last measure - 24 month |
| Peripheral arterial disease | ICPC = K92 |
| Coronary heart disease | ICPC = K74\|K75\|K76 |
| Chronic heart failure | ICPC = K77 |
| Stroke | ICPC = K90\|K91 |
| Retinopathy | ICPC = F83 |
| Neuropathy | ICPC = N94 |
| Insulin | ATC = 'A10A' |
| Oral | ATC = 'A10B' |
| Antihypertensive | ATC = 'C02\|C03A\|C03EA01\|C0[78]\|C09[AB]' |
| antithrombotic agents | ATC = 'B01A' AND ATC not = '^B01AD' |
| lipid lowering | ATC = 'C10' |

|  | ***Type*** | ***Subject*** | **Variable** | **OR** | **95% CI** | **p-value** |
| --- | --- | --- | --- | --- | --- | --- |
| **Primary outcomes** | Clinical QI | BP | GP gender (ref = female) | 0.654 | 0.448 - 0.955 | <0.05 |
|  |  |  | GP age | 1.018 | 0.999 - 1.037 | 0.065 |
|  |  |  | Volume of patients with diabetes | 1.051 | 0.971 - 1.138 | 0.22 |
|  |  |  | Main effect intervention | 0.939 | 0.646 - 1.364 | 0.742 |
|  |  |  | Main effect time | 0.992 | 0.974 - 1.01 | 0.38 |
|  |  |  | Interactive effect time*intervention | 1.026 | 0.999 - 1.053 | 0.06 |
|  | Process QI | HbA1c | GP gender (ref = female) | 0.903 | 0.662 - 1.231 | 0.518 |
|  |  |  | GP age | 0.985 | 0.970 – 1.000 | 0.050 |
|  |  |  | Volume of patients with diabetes | 1.004 | 0.938 - 1.075 | 0.902 |
|  |  |  | Main effect intervention | 0.736 | 0.527 - 1.029 | 0.073 |
|  |  |  | Main effect time | 0.983 | 0.961 - 1.006 | 0.144 |
|  |  |  | Interactive effect time*intervention | 1.014 | 0.982 - 1.047 | 0.389 |
| **Secondary outcomes** | Process QI | BP | GP gender (ref = female) | 0.646 | 0.395 - 1.056 | 0.081 |
|  |  |  | GP age | 1.017 | 0.992 - 1.041 | 0.178 |
|  |  |  | Volume of patients with diabetes | 1.086 | 0.974 - 1.211 | 0.136 |
|  |  |  | Main effect intervention | 1.194 | 0.726 - 1.962 | 0.485 |
|  |  |  | Main effect time | 0.986 | 0.966 - 1.007 | 0.193 |
|  |  |  | Interactive effect time*intervention | 1.036 | 1.004 - 1.070 | <0.05 |
|  | Clinical QI | HbA1c | GP gender (ref = female) | 0.810 | 0.592 - 1.107 | 0.185 |
|  |  |  | GP age | 0.990 | 0.975 - 1.005 | 0.200 |
|  |  |  | Volume of patients with diabetes | 1.041 | 0.968 - 1.118 | 0.278 |
|  |  |  | Main effect intervention | 0.729 | 0.512 - 1.039 | 0.08 |
|  |  |  | Main effect time | 0.990 | 0.971 - 1.009 | 0.284 |
|  |  |  | Interactive effect time*intervention | 1.023 | 0.995 - 1.051 | 0.106 |
|  | Process QI | Chol | GP gender (ref = female) | 0.992 | 0.641 - 1.535 | 0.972 |
|  |  |  | GP age | 0.989 | 0.969 - 1.010 | 0.298 |
|  |  |  | Volume of patients with diabetes | 1.087 | 0.984 - 1.200 | 0.100 |
|  |  |  | Main effect intervention | 0.725 | 0.448 - 1.175 | 0.192 |
|  |  |  | Main effect time | 0.993 | 0.976 - 1.011 | 0.461 |
|  |  |  | Interactive effect time*intervention | 1.027 | 1.000 - 1.055 | <0.05 |
|  | Clinical QI | Chol | GP gender (ref = female) | 1.000 | 0.678 - 1.475 | 0.998 |
|  |  |  | GP age | 0.991 | 0.972 - 1.010 | 0.337 |
|  |  |  | Volume of patients with diabetes | 1.096 | 0.995 - 1.206 | 0.063 |
|  |  |  | Main effect intervention | 0.791 | 0.475 - 1.316 | 0.367 |
|  |  |  | Main effect time | 0.995 | 0.977 - 1.014 | 0.602 |
|  |  |  | Interactive effect time*intervention | 1.010 | 0.983 - 1.038 | 0.471 |

Supplementary table 4: Detailed results of logistic regression models for primary and secondary outcomes

Supplementary table 5: Random effects of hierarchical logistic model

| **QI** | **Cluster** | **Variance** | **Std. deviation** |
| --- | --- | --- | --- |
| bp_proc_qual | arzt_id:praxis_id | 0.57 | 0.76 |
| bp_proc_qual | praxis_id | 0.20 | 0.44 |
| bp_out_qual | arzt_id:praxis_id | 0.36 | 0.60 |
| bp_out_qual | praxis_id | 0.08 | 0.28 |
| hba1c_proc_qual | arzt_id:praxis_id | 0.18 | 0.43 |
| hba1c_proc_qual | praxis_id | 0.12 | 0.34 |
| hba1c_out_qual | arzt_id:praxis_id | 0.18 | 0.42 |
| hba1c_out_qual | praxis_id | 0.17 | 0.41 |
| chol_proc_qual | arzt_id:praxis_id | 0.36 | 0.60 |
| chol_proc_qual | praxis_id | 0.33 | 0.57 |
| chol_out_qual | arzt_id:praxis_id | 0.23 | 0.48 |
| chol_out_qual | praxis_id | 0.49 | 0.70 |

Supplementary table 6: Baseline characteristics and baseline quality indicator performance compared by group

|  | **Intervention group** | **Control group** | **Non-participants** |
| --- | --- | --- | --- |
| **Practice and GP characteristics** |  |  |  |
| Practices | 21 | 22 | 16 |
| GPs | 36 | 35 | 33 |
| GPs per practice | 1.0 (1.0 - 2.0) | 1.0 (1.0 - 2.0) | 1.0 (1.0 - 2.0) |
| Network participation (% no) | 4 (11.1) | 2(5.7) | 11 (33.3) |
| Age | 48.0 (42.0 - 57.3) | 54.0 (46.0 - 63.0) | 51.5 (43.8 - 54.3) |
| Gender (% male) | 24 (66.7) | 27 (77.1) | 21 (63.6) |
| Volume of patients with diabetes per GP (%) | 5.0 (3.3 - 6.4) | 4.9 (3.7 - 6.4) | 4.1 (2.9 - 6.4) |
| **Patient characteristics** |  |  |  |
| Patients | 1771 | 2067 | 1541 |
| Patient age | 70.0 (59.0 - 79.0) | 69.0 (60.0 - 77.0) | 68.0 (58.0 - 77.0) |
| Patient gender (% male) | 989 (55.8) | 1209 (58.6) | 900 (58.4) |
| **Treatment and disease characteristics** |  |  |  |
| Consultations in observation period | 8 (3 - 15) | 7 (3 - 12) | 7 (3 - 15) |
| Blood pressure measurements | 3 (1 - 4) | 2 (1 - 4) | 2 (1 - 4) |
| Systolic blood pressure [mm Hg] | 137.5 (128.4 - 149.0) | 134.0 (125.0 - 143.3) | 136.0 (127.4 - 148.0) |
| Diastolic blood pressure [mm Hg] | 80.0 (74.0 - 86.0) | 80.0 (73.3 - 85.0) | 81.0 (75.0 - 88.0) |
| HbA1c measurements | 2 (1 - 3) | 3 (1 - 4) | 2(1 - 3) |
| HbA1c [%] | 6.8 (6.3 - 7.5) | 6.8 (6.3 - 7.5) | 6.8 (6.4 - 7.5) |
| Cholesterol measurement | 1 (1 - 2) | 1 (1- 1) | 1 (1 - 2) |
| Cholesterol [mmol/l] | 4.5 (3.8 - 5.3) | 4.6 (3.8 - 5.4) | 4.5 (3.9 - 5.3) |
| BMI measurements | 2 (1 - 3) | 2 (1 - 3) | 2 (1 - 3) |
| BMI [kg/m^2^] | 29.4 (26.5 - 33.1) | 29.6 (26.3 - 33.5) | 29.8 (26.3 - 34.2) |
| **Diabetes associated Comorbidities** |  |  |  |
| Obesity | 759 (42.9) | 855 (41.4) | 392 (25.4) |
| Hypertension | 1566 (88.4) | 1765 (85.4) | 1288 (83.6) |
| Hyperlipidaemia | 1098 (62.0) | 1167 (56.5) | 665 (43.2) |
| Chronic kidney disease | 212 (12.0) | 242 (11.7) | 121 (7.9) |
| Peripheral arterial disease | 54 (3.0) | 80 (3.9) | 16 (1.0) |
| Coronary heart disease | 127 (7.2) | 107 (5.2) | 55 (3.6) |
| Heart failure | 48 (2.7) | 35 (1.7) | 22 (1.4) |
| Stroke | 29 (1.6) | 38 (1.8) | 12 (0.8) |
| Retinopathy | 11 (0.6) | 3 (0.1) | 1 (0.1) |
| Neuropathy | 44 (2.5) | 56 (2.7) | 8 (0.5) |
| **Diabetes associated medication** |  |  |  |
| Insulin only | 159 (9.0) | 129 (6.2) | 119 (7.7) |
| Oral anti-diabetic medication only | 866 (48.9) | 1112 (53.8) | 961 (62.4) |
| Combination therapy insulin and oral anti-diabetic medication | 258 (14.6) | 262 (12.7) | 229 (14.9) |
| Antihypertensive medication | 862 (48.7) | 975 (47.2) | 738 (47.9) |
| Antiplatelet therapy and anticoagulants | 736 (41.6) | 806 (39.0) | 661 (42.9) |
| Lipid lowering medication | 258 (14.6) | 262 (12.7) | 656 (42.6) |

GP: general practitioner; BMI: body mass index;

Supplementary table 7: Detailed results of logistic regression models for primary and secondary outcomes for the additional analysis

|  | ***Type*** | ***Subject*** | **Variable** | **OR** | **95% CI** | **p-value** |
| --- | --- | --- | --- | --- | --- | --- |
| **Primary outcomes** | Clinical QI | BP | GP gender (ref = female) | 0.74 | 0.54-1.01 | 0.054 |
|  |  |  | GP age | 1.02 | 1.00-1.04 | <0.05 |
|  |  |  | Volume of patients with diabetes | 1.06 | 0.99-1.13 | 0.08 |
|  |  |  | Main effect intervention «non-participants» | 0.70 | 0.48-1.03 | 0.073 |
|  |  |  | Main effect intervention «treat» | 0.98 | 0.66-1.44 | 0.905 |
|  |  |  | Main effect time | 0.99 | 0.97-1.01 | 0.383 |
|  |  |  | Interactive effect time*intervention «non-participants» | 1.01 | 0.98-1.04 | 0.563 |
|  |  |  | Interactive effect time*intervention «treat» | 1.03 | 1.00-1.05 | 0.06 |
|  | Process QI | HbA1c | GP gender (ref = female) | 0.82 | 0.64-1.07 | 0.14 |
|  |  |  | GP age | 0.99 | 0.97-1.00 | 0.056 |
|  |  |  | Volume of patients with diabetes | 1.04 | 0.98-1.09 | 0.206 |
|  |  |  | Main effect intervention «non-participants» | 0.81 | 0.57-1.15 | 0.23 |
|  |  |  | Main effect intervention «treat» | 0.72 | 0.52-0.99 | <0.05 |
|  |  |  | Main effect time | 0.98 | 0.96-1.01 | 0.145 |
|  |  |  | Interactive effect time*intervention «non-participants» | 0.99 | 0.96-1.03 | 0.638 |
|  |  |  | Interactive effect time*intervention «treat» | 1.01 | 0.98-1.05 | 0.391 |
| **Secondary outcomes** | Process QI | BP | GP gender (ref = female) | 0.70 | 0.47-1.03 | 0.068 |
|  |  |  | GP age | 1.02 | 1.00-1.05 | <0.05 |
|  |  |  | Volume of patients with diabetes | 1.09 | 1.01-1.18 | <0.05 |
|  |  |  | Main effect intervention «non-participants» | 0.85 | 0.52-1.38 | 0.499 |
|  |  |  | Main effect intervention «treat» | 1.27 | 0.79-2.04 | 0.321 |
|  |  |  | Main effect time | 0.99 | 0.97-1.01 | 0.193 |
|  |  |  | Interactive effect time*intervention «non-participants» | 0.99 | 0.96-1.02 | 0.476 |
|  |  |  | Interactive effect time*intervention «treat» | 1.04 | 1.00-1.07 | <0.05 |
|  | Clinical QI | HbA1c | GP gender (ref = female) | 0.82 | 0.63-1.05 | 0.113 |
|  |  |  | GP age | 0.99 | 0.98-1.00 | 0.155 |
|  |  |  | Volume of patients with diabetes | 1.07 | 1.01-1.13 | <0.05 |
|  |  |  | Main effect intervention «non-participants» | 0.83 | 0.58-1.19 | 0.31 |
|  |  |  | Main effect intervention «treat» | 0.72 | 0.52-1.00 | 0.053 |
|  |  |  | Main effect time | 0.99 | 0.97-1.01 | 0.286 |
|  |  |  | Interactive effect time*intervention «non-participants» | 1.00 | 0.97-1.03 | 0.872 |
|  |  |  | Interactive effect time*intervention «treat» | 1.02 | 1.00-1.05 | 0.105 |
|  | Process QI | Chol | GP gender (ref = female) | 0.96 | 0.64-1.43 | 0.83 |
|  |  |  | GP age | 0.99 | 0.97-1.01 | 0.263 |
|  |  |  | Volume of patients with diabetes | 1.06 | 0.97-1.15 | 0.219 |
|  |  |  | Main effect intervention «non-participants» | 0.66 | 0.36-1.22 | 0.182 |
|  |  |  | Main effect intervention «treat» | 0.71 | 0.40-1.27 | 0.251 |
|  |  |  | Main effect time | 0.99 | 0.98-1.01 | 0.452 |
|  |  |  | Interactive effect time*intervention «non-participants» | 0.98 | 0.95-1.01 | 0.143 |
|  |  |  | Interactive effect time*intervention «treat» | 1.03 | 1.00-1.06 | <0.05 |
|  | Clinical QI | Chol | GP gender (ref = female) | 0.99 | 0.70-1.41 | 0.968 |
|  |  |  | GP age | 0.99 | 0.97-1.01 | 0.253 |
|  |  |  | Volume of patients with diabetes | 1.06 | 0.98-1.15 | 0.159 |
|  |  |  | Main effect intervention «non-participants» | 0.73 | 0.39-1.34 | 0.308 |
|  |  |  | Main effect intervention «treat» | 0.78 | 0.44-1.38 | 0.391 |
|  |  |  | Main effect time | 1.00 | 0.98-1.01 | 0.593 |
|  |  |  | Interactive effect time*intervention «non-participants» | 0.99 | 0.96-1.02 | 0.441 |
|  |  |  | Interactive effect time*intervention «treat» | 1.01 | 0.98-1.04 | 0.449 |

OR: odds ratio; CI: confidence interval; QI: quality indicator; BP: blood pressure; Chol: cholesterol; GP: General practitioner
